# iCLIP Predicts the Dual Splicing Effects of TIA-RNA Interactions

**DOI:** 10.1371/journal.pbio.1000530

**Published:** 2010-10-26

**Authors:** Zhen Wang, Melis Kayikci, Michael Briese, Kathi Zarnack, Nicholas M. Luscombe, Gregor Rot, Blaž Zupan, Tomaž Curk, Jernej Ule

**Affiliations:** 1 Medical Research Council (MRC) – Laboratory of Molecular Biology, Hills Road, Cambridge, United Kingdom; 2 European Molecular Biology Laboratory (EMBL) – European Bioinformatics Institute, Wellcome Trust Genome Campus, Hinxton, United Kingdom; 3 EMBL, Genome Biology Unit, Heidelberg, Germany; 4 Faculty of Computer and Information Science, University of Ljubljana, Ljubljana, Slovenia; National Cancer Institute, United States of America

## Abstract

The regulation of alternative splicing involves interactions between RNA-binding proteins and pre-mRNA positions close to the splice sites. T-cell intracellular antigen 1 (TIA1) and TIA1-like 1 (TIAL1) locally enhance exon inclusion by recruiting U1 snRNP to 5′ splice sites. However, effects of TIA proteins on splicing of distal exons have not yet been explored. We used UV-crosslinking and immunoprecipitation (iCLIP) to find that TIA1 and TIAL1 bind at the same positions on human RNAs. Binding downstream of 5′ splice sites was used to predict the effects of TIA proteins in enhancing inclusion of proximal exons and silencing inclusion of distal exons. The predictions were validated in an unbiased manner using splice-junction microarrays, RT-PCR, and minigene constructs, which showed that TIA proteins maintain splicing fidelity and regulate alternative splicing by binding exclusively downstream of 5′ splice sites. Surprisingly, TIA binding at 5′ splice sites silenced distal cassette and variable-length exons without binding in proximity to the regulated alternative 3′ splice sites. Using transcriptome-wide high-resolution mapping of TIA-RNA interactions we evaluated the distal splicing effects of TIA proteins. These data are consistent with a model where TIA proteins shorten the time available for definition of an alternative exon by enhancing recognition of the preceding 5′ splice site. Thus, our findings indicate that changes in splicing kinetics could mediate the distal regulation of alternative splicing.

## Abstract 2

Transcriptome-wide analysis of protein-RNA interactions predicts the dual splicing effects of TIA proteins, showing that their local enhancing function is associated with diverse distal splicing silencing effects.

## Author Summary

Studies of splicing regulation have generally focused on RNA elements located close to alternative exons. Recently, it has been suggested that splicing of alternative exons can also be regulated by distal regulatory sites, but the underlying mechanism is not clear. The TIA proteins are key splicing regulators that enhance the recognition of 5′ splice sites, and their distal effects have remained unexplored so far. Here, we use a new method to map the positions of TIA-RNA interactions with high resolution on a transcriptome-wide scale. The identified binding positions successfully predict the local enhancing and distal silencing effects of TIA proteins. In particular, we show that TIA proteins can regulate distal alternative 3′ splice sites by binding at the 5′ splice site of the preceding exon. This result suggests that alternative splicing is affected by the timing of alternative exon definition relative to the recognition of the preceding 5′ splice site. These findings highlight the importance of analysing distal regulatory sites in order to fully understand the regulation of alternative splicing.

## Introduction

Pre-mRNA splicing is catalysed by small nuclear ribonucleoprotein particles (snRNP) that recognise the splice sites on pre-mRNA and remove the introns with great precision. U1 and U2 snRNPs recognise the core motifs present at the 5′ and 3′ splice sites, respectively [1]. These core splice site motifs, however, contain only about half of the information required to define exon/intron boundaries [2]. Additional sequence elements can recruit regulatory RNA-binding proteins either to enhance or silence splice site recognition depending on their position relative to the splice sites [3],[4].

T-cells intracellular antigen 1 (TIA1) and TIA1-like1 (TIAL1, also known as TIAR) are closely related RNA-binding proteins. They have three RNA recognition motifs (RRMs) and a carboxyl-terminal glutamine-rich region [5],[6]. RRM2 is the major domain binding to uridine-rich sequences, RRM3 is thought to bind to RNA with no specificity, and RRM1 has no detectable RNA binding affinity in vitro [7]. Instead, RRM1 and the C-terminus interact with U1 snRNP to enhance its recruitment to the 5′ splice site of alternative exons [8]–[11].

TIA1 and TIAL1 are involved in multiple aspects of RNA metabolism. They are present in both the cytoplasm and the nucleus and shuttle between these two compartments in a manner that requires the RRM2 and RRM3 domains [12],[13]. In the nucleus, TIA1 and TIAL1 regulate alternative splicing by binding to U-rich sequences adjacent to the 5′ splice site and recruiting U1-C to promote exon inclusion [8],[10],[11],[14],[15]. They also regulate the splicing of their own mRNAs, and the resulting two major isoforms have different splicing activity [16],[17]. In the cytoplasm, TIA1 and TIAL1 function as translational silencers by binding to the 3′ untranslated region (3′ UTR) of mRNAs [18],[19]. They were also implicated in stress-induced translational silencing in stress granules [12],[20]. In addition, TIA1 and TIAL1 were shown to promote apoptosis [21], and depletion of both proteins promotes cell proliferation [22].

The role of *cis*-regulatory RNA motifs located close to alternative exons has been widely investigated, but recent studies suggest that distal regulatory motifs might also play an important role [4],[23],[24]. For instance, Nova1 and Nova2 proteins can silence inclusion of an alternative exon when binding downstream of the preceding exon [4],[24]. In contrast, Nova proteins enhance inclusion when binding directly downstream of an alternative exon [4],[24]. This suggested that the local and distal effects of Nova binding downstream of a 5′ splice site are reciprocal [25]. Since the function of TIA proteins in recruiting U1 snRNP to 5′ splice site is well characterised, these proteins offered a unique opportunity for a comprehensive study of the distal splicing effects of changes in 5′ splice site recognition.

Ultraviolet (UV)-crosslinking and immunoprecipitation (CLIP) was first developed to identify RNA sites bound by the splicing regulators Nova1 and Nova2 in brain tissue [26]. The traditional CLIP cDNA library preparation protocol suffers from a potential loss of cDNAs due to truncation immediately before the “crosslink site,” where at least one amino acid remains covalently attached after proteinase K digestion [27]. Therefore, we used a modified cDNA library preparation protocol that was recently developed (iCLIP), which identifies truncated cDNAs by introducing the second adapter to cDNAs after reverse transcription [28]. In addition, iCLIP introduces a random DNA sequence (barcode) to cDNAs during reverse transcription to differentiate between unique cDNA products and PCR duplicates. Since the first nucleotide of resulting cDNA sequences most likely locates directly downstream of the crosslink site, iCLIP enables quantitative and high resolution analysis of protein crosslinking to the target RNAs [28].

Here, we used iCLIP to identify the RNA crosslink sites of TIA proteins. iCLIP showed a high density of TIA crosslinking in 3′ UTRs of mRNAs and in non-coding RNAs (ncRNAs). Intronic TIA binding clusters were restricted to positions immediately downstream of 5′ splice sites. TIA binding at the 5′ splice site of an alternative exon and/or the preceding exon predicted its dual splicing effects. TIA binding enhanced inclusion of proximal upstream alternative exons and usage of upstream alternative splice sites but silenced distal downstream alternative exons if these lacked direct TIA binding. Interestingly, TIA proteins also regulated distal alternative 3′ splice sites, suggesting that by enhancing 5′ splice site recognition, they can indirectly silence downstream alternative exons.

## Results

### iCLIP and iCLAP Identify Crosslink Sites of TIA1 or TIAL1

**Figure 1:**
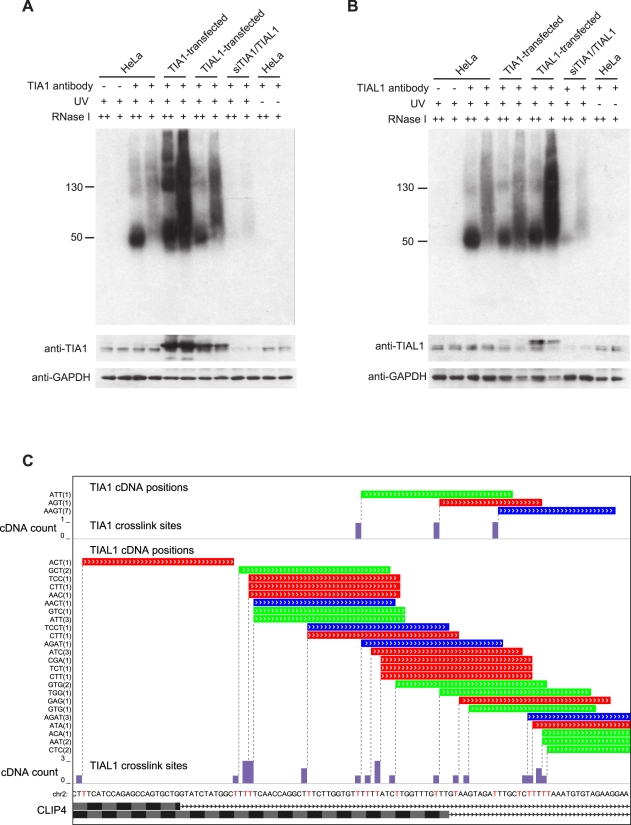
iCLIP identifies the TIA1 and TIAL1 crosslink sites with nucleotide resolution. Autoradiogram of ^32^P-labelled RNA crosslinked to TIA1 (A) or TIAL1 (B) in HeLa cells. Immunoprecipitation was performed with either anti-TIA1 or anti-TIAL1 antibody using lysate from UV-crosslinked HeLa cells, cells transfected with TIA1 or TIAL1, TIA1/TIAL1 KD cells, or non-crosslinked cells. High and low RNase concentrations were used and protein G beads were used as a control. The Western blots below the autoradiograms show the input lysate used for each immunoprecipitation. (C) TIA1 and TIAL1 crosslink to uridine tracts downstream of the alternative 5′ splice sites in the CLIP4 gene. The cDNA positions are colour-coded for three replicate TIA1 and TIAL1 experiments, and the random barcode (shown on the left) is used to identify unique iCLIP cDNAs (number in brackets indicates the number of corresponding PCR duplicates). Below, the bar graphs show the cDNA count (number of cDNAs at each crosslink site). Pre-mRNA sequence is shown below with crosslink nucleotides in red. The exon and intron positions of the two isoforms of CLIP4 mRNA are shown at the bottom.

iCLIP was used to identify the crosslink sites of TIA1 and TIAL1 in HeLa cells in a transcriptome-wide manner. Briefly, cells were UV-irradiated, lysed, and RNA was digested with RNase I to a size of approximately 40–100 nucleotides (nt). The proteins were immunoprecipitated using TIA1 or TIAL1-specific antibodies, and the protein-RNA complexes were subjected to 5′ labelling using ^32^P-γ-ATP for visualisation after SDS-PAGE separation. The specificity of each antibody was determined by overexpression of TIA1 or TIAL1 in HeLa cells (Figures 1A, 1B, S1A and S1B). TIA1 and TIAL1 antibodies each detected RNA-protein complexes of correct size only from UV-crosslinked cells and only if an antibody was used for immunoprecipitation. Furthermore, the signal decreased in TIA1/TIAL1 double knockdown (KD) cells and increased when the corresponding protein was overexpressed (Figure 1A and 1B). There was a slight cross-reactivity of the TIA1 antibody to the TIAL1 protein (Figures 1A and S1A, when TIAL1 was overexpressed). However, during immunoprecipitation, the TIA1 antibody mainly recognised the TIA1 protein, as no increase was seen when TIAL1 was overexpressed (Figure S1B).

To assess the RNA sequence specificity of TIA1 or TIAL1 without the use of antibodies, we also developed crosslinking and affinity purification (iCLAP), a method to purify Strep/His double-tagged TIA1 and TIAL1 proteins using stringent affinity purification. This method circumvents any cross-reactivity of antibody that would identify the same crosslink sites for both proteins. TIA1 and TIAL1 with the Strep/His tag on the N- or C-terminus were overexpressed in HeLa cells. After UV crosslinking and RNase I digestion, the protein-RNA complexes were first purified with magnetic streptavidin bead before ligation to the 3′ RNA adaptor. Cobalt beads were then used to further purify the protein-RNA complexes under denaturing conditions (8 M urea, Figure S1E). iCLAP detected protein-RNA complexes only if cells were transfected with an appropriate construct, and no signal was detected in vector-transfected or non-crosslinked cells (Figure S1F). In summary, the analysis of radioactive protein-RNA complexes indicated that both iCLIP and iCLAP isolated specific protein-RNA complexes without contamination from other co-purified proteins or RNAs.

To amplify the co-purified RNAs, these were dephosphorylated and ligated to the 3′ RNA adaptor on beads during immunoprecipitation (iCLIP) or affinity purification (iCLAP). After SDS-PAGE and nitrocellulose transfer, the protein-RNA complexes of 70–150 kDa were excised from the membrane (Figure S1C) and subjected to proteinase K digestion. The RNA was reverse transcribed with a primer complementary to the 3′ RNA adaptor, which contained a second half complementary to the 5′ Solexa sequencing primer separated by a BamHI digestion site. The cDNA was then self-circularised, digested with BamHI, giving a product with corresponding adaptors at both ends, and amplified by PCR (Figure S1D). PCR products were sequenced using single-end 44 nt reads on the Illumina GA2 system.

Three independent replicate iCLIP experiments and one iCLAP experiment were performed for both TIA1 and TIAL1 (Table S1). In total, 18.4 million iCLIP sequences were generated, 74% of which aligned to the human genome by allowing only single genomic hits and one nucleotide mismatch (Table S2). Unique cDNAs were identified based on random barcodes, and the crosslink site was mapped to the first nucleotide preceding the start of the cDNAs (Figure 1C). Together, iCLIP produced 869,782 unique cDNA reads for TIA1 and 2,966,801 unique cDNA reads for TIAL1 (Table S2). The iCLIP no-antibody controls, performed in parallel with two of the iCLIP experiments, did not generate detectable PCR products. When submitted for sequencing, they generated 1,074 and 7,798 unique cDNAs mapping to the human genome (Table S2). Since TIA1 or TIAL1 iCLIP generated 100-fold more cDNAs than controls, we estimated that over 99% of cDNAs from the iCLIP experiment represent RNA sites specifically crosslinked to TIA1 or TIAL1.

### TIA1 and TIAL1 Bind the Same RNA Sites

**Figure 2:**
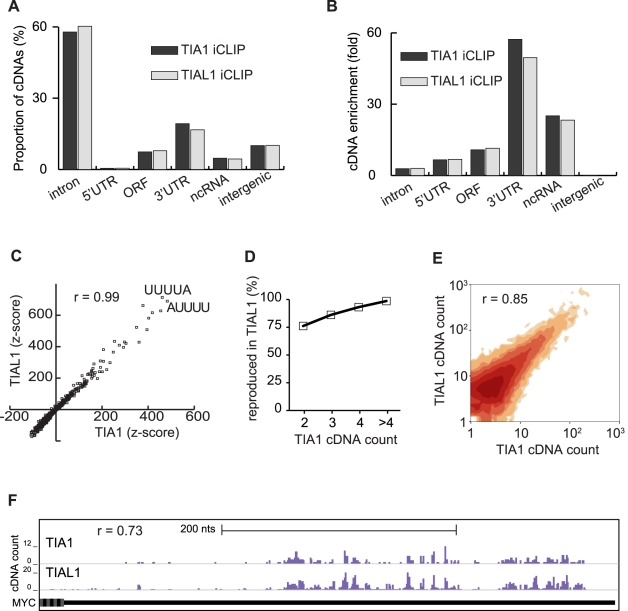
TIA1 and TIAL1 crosslink to the same positions in human RNAs. (A) The percentage of cDNAs from TIA1 and TIAL1 iCLIP that mapped to different types of RNAs. (B) The fold enrichment of average cDNA density from TIA1 and TIAL1 iCLIP in different types of RNAs relative to the average cDNA density in the whole genome. (C) Pentamer *z* scores at the 21 nt sequence surrounding crosslink sites (−10 nt to +10 nt) are shown for TIA1 and TIAL1 iCLIP. The sequences of the two most enriched pentamers and the Pearson correlation coefficient (*r*) between the TIA1 and TIAL1 *z* scores are shown. (D) Reproducibility of TIA1 and TIAL1 crosslink clusters. Percentage of crosslink clusters with a given cDNA count in TIA1 iCLIP that were also identified in TIAL1 iCLIP is shown. (E) Contour plot comparing TIA1 and TIAL1 cDNA counts in the 46,970 crosslink clusters. The darkness of contours increases with the number of clusters. The Pearson correlation coefficient (*r*) between the TIA1 and TIAL1 cDNA counts in the clusters is shown. (F) Crosslink sites of TIA1 and TIAL1 in the 3′ UTR of the MYC gene. The bar graph shows the number of cDNAs that identified each crosslink site. The Pearson correlation coefficient (*r*) between the TIA1 and TIAL1 cDNA counts at individual nucleotides is shown.

The random barcode introduced into iCLIP cDNAs allowed us to analyse the distribution of TIA1 and TIAL1 on human RNAs in a quantitative and reproducible manner (Figure S2). Only 1.7% of cDNAs mapped in antisense orientation to annotated genes, confirming the high strand specificity of iCLIP. Only 10% of cDNAs mapped to intergenic regions (Figure 2A). The highest cDNA density was seen in 3′ UTRs and ncRNAs, which together contained 22% of all cDNAs (Figure 2A and 2B). 2,277 ncRNAs and 8,602 3′ UTRs had a higher cDNA density than the whole-genome average, and the cDNA enrichment correlated between TIA1 and TIAL1 iCLIP (Pearson correlation coefficient *r* = 0.95 and *r* = 0.90, respectively; Figure S3G and S3H). The ncRNA and 3′ UTR sites with the highest cDNA counts mapped to highly expressed RNAs such as tRNAs and histone mRNAs (Figure S4B). Interestingly, cDNA enrichment in 3′ UTRs was 5-fold higher than in the coding sequence (Figure 2B), in agreement with past findings that TIA proteins bind 3′ UTR to regulate translation [18],[29]–[31].

Fifty-eight percent of TIA1 and 60% of TIAL1 cDNAs mapped to introns (Figure 2A). 67,002 introns had a cDNA density higher than the whole-genome average, and the cDNA enrichment correlated between TIA1 and TIAL1 iCLIP (*r* = 0.81; Figure S3I). The cDNA density in introns was on average 18-fold lower than in 3′ UTRs and ncRNAs (Figures 2B and S3I). Past studies have shown that TIA1 and TIAL1 regulate alternative splicing of exon 6 of FAS mRNA [14],[17],[32],[33]. Both TIA1 and TIAL1 crosslinked to previously characterised intronic binding sites in FAS pre-mRNA (Figure S5A).

Past studies suggested that TIA1 and TIAL1 have different RNA binding specificities [7],[34]. However, the two proteins can regulate alternative splicing of the same exons [17]. We therefore analysed the in vivo RNA specificity of the two proteins using our iCLIP data. As a control, the iCLIP positions were randomised within the co-expressed genomic regions. The 21 nt sequence surrounding the crosslink sites was compared to randomised positions to identify the pentamers enriched at the TIA1 and TIAL1 crosslink sites. Pentamer enrichment in TIA1 and TIAL1 iCLIP data was highly correlated, and UUUUA and AUUUU were the two most common pentamers (*r* = 0.99; Figures 2C and S3A, Table S3). Comparing replicate iCLIP experiments of either protein verified the high reproducibility of the observed sequence specificity (Figure S3C and S3D). Similarly, iCLAP experiments with both proteins were also highly correlated and were enriched for the same pentamers as iCLIP, independently supporting the determined sequence specificity of both proteins (Figure S3B). These results demonstrated that TIA1 and TIAL1 share the same in vivo RNA binding specificity.

Due to the high stringency of purification of protein-RNA complexes, iCLIP purified RNA sites that directly interact with TIA proteins. However, it is possible that some of these sites represent transient and low-affinity TIA-RNA interactions. To specifically analyse the high-affinity RNA binding sites, we determined clusters of TIA1 or TIAL1 crosslink sites with a maximum spacing of 15 nt containing a significant cDNA count when compared to randomised positions (FDR <0.05). This identified 12,048 TIA1 and 34,058 TIAL1 crosslink clusters. Uridine represented 82% of TIA1 and 75% of TIAL1 clustered crosslink sites (i.e., crosslink sites that located within these clusters) and was also the most common nucleotide at all positions up to 10 nt away from these crosslink sites (Figure S3E). This agreed with the past studies showing that TIA proteins bind to uridine-rich motifs [8],[14],[17],[19].

To compare the overlap between binding of TIA1 and TIAL1 to the same sites, we analysed the proportion of crosslink clusters identified by both proteins. Eighty-three percent (10,021 / 12,048) of TIA1 crosslink clusters overlapped with a TIAL1 crosslink site, and 59% (20,047 / 34,058) of TIAL1 crosslink clusters overlapped with a TIA1 crosslink site. The overlap depended on the number of cDNAs that defined a cluster. Ninety-nine percent of the crosslink clusters that were defined by five or more TIA1 cDNAs overlapped with a TIAL1 crosslink site (Figure 2D). We also assessed the distances between clustered TIA1 and TIAL1 crosslink sites. TIAL1 crosslink sites overlapped with TIA1 crosslink sites 15-fold more common than with randomised TIA1 iCLIP positions (Figure S3F). These analyses indicated that the binding sites of TIA1 and TIAL1 largely overlap.

Since both proteins showed a redundant binding behaviour, the iCLIP data of TIA1 and TIAL1 were merged to increase the reliability of cluster definition, which depends on the number of unique cDNA sequences. 46,970 crosslink clusters were identified in the joint TIA1/TIAL1 data with a maximum spacing of 15 nt (FDR<0.05). The cDNA counts within these crosslink clusters were highly correlated between TIA1 and TIAL1 data, indicating that they had similar affinity to their common RNA binding sites (*r* = 0.85; Figure 2E). Furthermore, the cDNA counts were correlated even at the single-nucleotide level, as evident in the 3′ UTR of MYC mRNA, which is a functionally validated translational target of the TIA proteins (*r* = 0.73; Figures 2F and S5B) [30],[35]. Taken together, iCLIP data suggested that TIA1 and TIAL1 have similar affinity for their common RNA binding sites.

### iCLIP Predicts TIA Effects on Splicing of Cassette Exons

**Figure 3:**
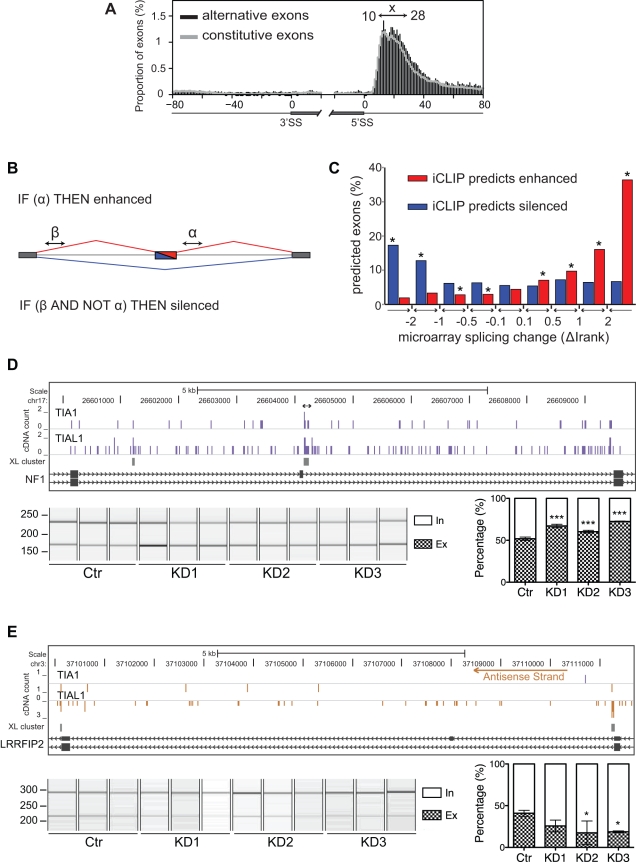
iCLIP predicts the regulation of alternative cassette exons. (A) The nucleotide-resolution RNA map of TIA1/TIAL1 iCLIP crosslink clusters at 5′ splice sites of constitutive (grey line) and alternative (black bars) exons. Percentage of exons containing crosslink sites in 20 nt of exonic and 80 nt of intronic sequence is shown. The region “x” 10–28 nt downstream of exon/intron boundaries contains 30-fold enrichment of crosslink events if compared by the last 20 nucleotides of exonic sequence. (B) Logic functions of the TIA iCLIP code that predict splicing regulation. α and β are regions 10–28 nt downstream of exon/intron boundaries that predict enhanced or silenced exons. (C) The exons analysed by the splice-junction microarray are divided into intervals relative to their splicing change, and percentage of exons predicted by iCLIP in each interval is shown. The stars mark those categories where predictions perform significantly better than on control exons (* *p*<0.05, Fisher's Exact Test). (D,E) iCLIP crosslink sites surrounding the enhanced exon 23a in NF1 pre-mRNA (D) and the silenced exon in LRRFIP2 pre-mRNA (E). The cDNA counts for TIA1 and TIAL1 are shown in bar graphs (blue bars represent crosslinking to the sense strand, and orange bars to the antisense strand of the genome), and the crosslink (XL) clusters (FDR<0.05) are marked with grey rectangles. The arrow above the bar graphs shows the previously identified TIA binding sites. RNA from KD cells prepared with three different siRNA oligonucleotides and their quantification was analysed by RT-PCR and capillary electrophoresis. Capillary electrophoresis image and signal quantification are shown below the bar graphs. Quantified transcripts including (in) or excluding (ex) the regulated alternative exon are marked on the right. Average quantification values of exon inclusion (white) and exclusion (grey) are given as a fraction of summed values. Error bars represent standard deviation of three replicates and *p* values are also calculated (* *p*<0.05, *** *p*<0.001, one-way ANOVA).

In order to identify the candidate positions where TIA proteins bind to regulate splicing, we compared the distribution of TIA1/TIAL1 iCLIP clustered crosslink sites close to constitutive and alternative cassette exons (Figure 3A). Thirty-fold enrichment was seen at positions 10–28 nt downstream of exon/intron boundaries compared to the last 20 nt of both type of exons. Surprisingly, the constitutive and alternative exons showed a similar extent of crosslinking in this region. Approximately 5% of both types of exons contained a TIA crosslink cluster in this region.

Since TIA proteins were previously described as splicing enhancers [8],[14], we hypothesised that TIA crosslink clusters could be used to predict proximal enhanced exons (Figure 3B). Therefore, a regulatory logic was defined where TIA crosslink clusters 10–28 nt downstream of an alternative exon (region α in Figure 3B) predicted enhanced exon inclusion. This predicted 1,620 alternative cassette exons as enhanced by TIA proteins. Furthermore, since binding of Nova proteins downstream of the preceding exon could silence distal alternative exons [4], we defined a second regulatory logic for distal silencing. TIA crosslink clusters 10–28 nt downstream of the preceding exon (region β in Figure 3B) predicted silenced alternative exon inclusion if TIA crosslink clusters were absent in the region α. This predicted 1,962 alternative cassette exons as silenced by TIA proteins.

To assess the iCLIP predictions in an unbiased way, the splicing changes in TIA1/TIAL1 KD HeLa cells were analysed using a high-resolution splice-junction microarray. Microarray data were analysed with the ASPIRE 3 software [28]. The microarray detected splicing changes in 1,213 cassette exons (|ΔIrank|≥1), 46 of which were further assessed using reverse transcription and PCR (RT-PCR) and capillary electrophoresis (Text S1). RNA was isolated from cells treated with three different siRNA oligonucleotide pairs targeting TIA1 and TIAL1, and with control siRNA oligonucleotides (for knockdown efficiency, see Figure S6). Primer pairs generated a PCR product for 40 of the 46 tested splicing events, with 30 detecting two or more splicing isoforms. Among these, RT-PCR validated 86.7% (26/30) of the splicing changes, confirming the high accuracy of the microarray data (Figure S9A, Table S4).

We assessed the accuracy of iCLIP predictions by comparing them to the splicing changes identified by the microarray. Exons were divided into subsets according to the confidence of the detected splicing change (ΔIrank), and the number of exons correctly predicted by iCLIP was identified in each subset. iCLIP predicted approximately 5% of false positives in control exons (|ΔIrank|<0.1; Figure 3C). However, iCLIP predicted a significantly higher number of exons among those with a detectable splicing change (*p*<0.05, Fisher's Exact Test; Figure 3C). For these exons, iCLIP correctly predicted the direction of splicing change for 105 of 123 exons (85%), out of which 87 were enhanced (ΔIrank ≥2) and 18 were silenced (ΔIrank ≤−2). For example, a TIA crosslink cluster downstream of exon 23a in NF1 pre-mRNA located to a previously described functional TIA binding site [36], and it correctly predicted the enhancing effect of the TIA proteins (Figure 3D). In contrast, a TIA crosslink cluster downstream of the exon preceding the alternative exon in LRRFIP2 pre-mRNA correctly indicated the TIA-dependent silencing (Figure 3E).

**Figure 4:**
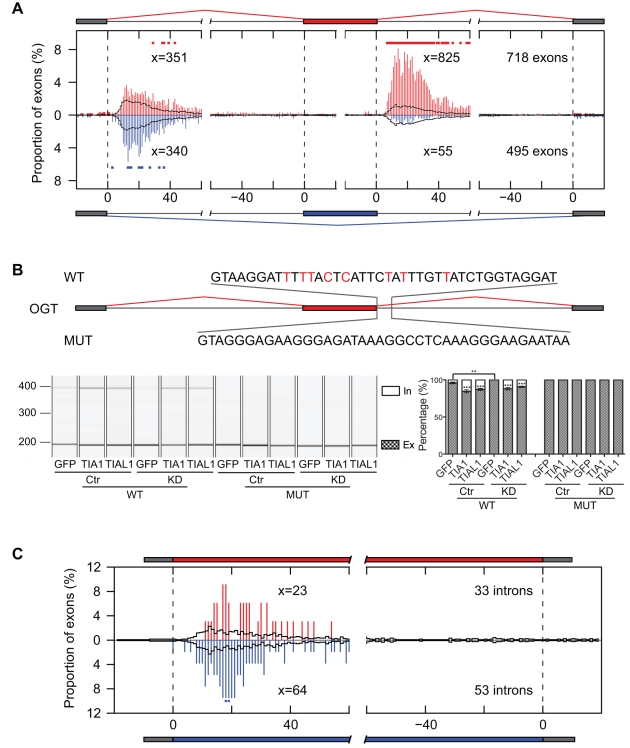
RNA maps of TIA-regulated cassette exons and introns. RNA map showing the percentage of alternative cassette exons (A) or retained introns (C) with clustered TIA1/TIAL1 crosslink sites at exon/intron boundaries, including 20 nt of exonic and 60 nt of intronic sequence. The number of crosslink events at each region “x” 10–28 nt downstream of exon/intron boundaries and the total number of exons analysed are shown for silenced (ΔIrank ≤1, blue bars), enhanced (ΔIrank ≥1, red bars), and control exons (|ΔIrank| ≤0.1, black line). The positions with a significantly higher number of crosslinking events in TIA-regulated RNAs than in control are indicated by red or blue dots above bars (* *p*<0.05, Fisher's Exact Test). (B) Minigene validation of enhanced alternative cassette exon from OGT1 pre-mRNA. The schematic diagram of each isoforms is shown, and the TIA binding sites are zoomed in above, with crosslinking nucleotides in red. The mutated sequences are shown below. Capillary electrophoresis image and signal quantification are shown below. Quantified transcripts including (in) or excluding (ex) the regulated alternative exon are marked on the right. Average quantification values of exon inclusion (white) and exclusion (grey) are given as a fraction of summed values. Error bars represent standard deviation of three replicates and *p* values are also calculated for TIA1 and TIAL1 overexpression compared to GFP transfected cells in either control or KD situation (** *p*<0.01, *** *p*<0.001, one-way ANOVA).

To assess whether TIA proteins bind at additional positions to regulate splicing, we comprehensively analysed the positions of TIA crosslinking in target RNAs with respect to the observed splicing changes (Figure 4A). Each clustered crosslink site in an individual RNA was considered as one crosslink event. The RNA map showed an increase in the number of crosslink events downstream of the exons, confirming that the predictive code included the primary positions where TIA proteins regulate splicing. There was a decrease in the number of crosslink events downstream of the silenced exons, combined with a significant increase downstream the preceding exon (Figure 4A). Similarly, mutually exclusive exons such as in FYN pre-mRNA displayed TIA crosslinking downstream of the enhanced exon but not in the vicinity of the silenced exon (Figure S8A). Since silenced exons had a reduced proximal TIA binding compared to control exons, TIA binding at the preceding exon was the most likely cause for the silencing effect.

The RNA map demonstrated a significant increase in the number of crosslink events downstream of the enhanced exons. To further validate the function of TIA binding at this position, we constructed a reporter minigene containing the alternative exon 5 and the flanking introns and exons of OGT pre-mRNA (Figure 4B). In the iCLIP data, the only TIA crosslink cluster present in this region was located downstream of the 5′ splice site. Overexpression of either TIA1 or TIAL1 in HeLa cells increased exon inclusion, whereas TIA KD HeLa cells decreased exon inclusion (Figure 4B). Overexpression of either TIA1 or TIAL1 in the KD cells was able to restore exon inclusion. This confirmed that both TIA1 and TIAL1 could enhance inclusion of the exon.

To directly test whether the RNA sequence underlying the TIA crosslink cluster is necessary for the ability of TIA proteins to enhance exon inclusion, the 40 nt of intronic sequence downstream of the exon were replaced with a sequence from CDC25C pre-mRNA, which did not contain any TIA crosslink sites (Figure 4B). Splicing of the mutant minigene did not change in response to the increased or decreased TIA protein levels (Figure 4B). Thus, the TIA crosslink clusters located downstream of enhanced exons identified the RNA sites necessary to mediate the enhancing effect of TIA proteins.

### TIA Proteins Maintain Splicing Fidelity at the 5′ Splice Site

Extensive TIA crosslinking downstream of constitutive exons suggested that in addition to regulating alternative splicing, TIA proteins might also play a role in maintaining splicing fidelity. Microarray analysis detected increased retention of 143 introns and decreased retention of 102 introns in TIA KD cells. We tested 18 of these introns using real-time PCR with a 94% validation rate (Figure S7B, Table S5). An example of an intron retained in KD cells is shown in PIAA2 pre-mRNA, which contains TIA crosslink sites downstream of the 5′ splice site (Figure S7A). The introns that were inefficiently spliced in KD cells had a significantly increased number of crosslink events downstream of the 5′ splice sites compared to control introns, indicating that TIA1 negatively regulates intron retention (Figure 4C). This is consistent with a past study using the msl-2 reporter minigene, which showed that TIA1 binding to the uridine-rich track prevents intron retention [37]. Our results indicate that maintaining splicing fidelity at 5′ splice sites of constitutive exons is a widespread function of TIA proteins.

### iCLIP Predicts TIA Effects on Usage of Alternative 5′ Splice Sites

**Figure 5:**
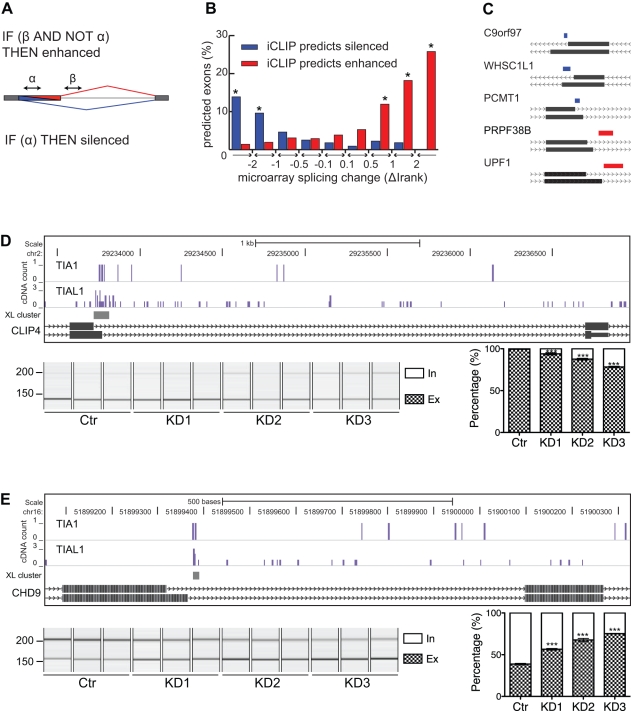
iCLIP predicts the regulation of alternative 5′ splice sites. (A) Logic functions of the TIA iCLIP code that predict splicing regulation. α and β are regions 10–28 nt downstream of exon/intron boundaries that predict enhanced or silenced exons. (B) The percentage of exons predicted by iCLIP is compared to the splicing change detected by microarray (* *p*<0.05, Fisher's Exact Test). (C) Diagrams showing a section of variable 5′ splice site isoforms of five pre-mRNAs (given on the left) predicted by iCLIP and validated by RT-PCR. The relative location of cDNA clusters is shown as a blue line at silenced and red line at enhanced variable exons. (D,E) iCLIP crosslink sites surrounding the enhanced variable-length exon in CLIP4 pre-mRNA (D) and silenced variable-length exon in CHD9 pre-mRNA (E). Depiction and labelling is as described in the legend for Figure 3D–E.

In addition to regulating splicing of cassette exons, TIA proteins can also regulate the usage of alternative 5′ splice sites [38]. We therefore hypothesised that TIA crosslink clusters could be used to predict regulation of variable-length exons (Figure 5A). Since the variable regions are often very short, precise identification of binding sites is crucial. To test whether the resolution of iCLIP was sufficient to resolve dual regulation of alternative 5′ splice sites, a regulatory logic was defined where TIA crosslink clusters 10–28 nt downstream of the intron-distal 5′ splice site (position α in Figure 5A) predicted silenced variable exons (i.e., increased usage of the intron-distal 5′ splice site). Conversely, TIA crosslink clusters 10–28 nt downstream of the intron-proximal 5′ splice site (position β in Figure 5A) predicted enhanced variable exons if TIA crosslink clusters were absent downstream of the intron-distal site. This logic predicted TIA-dependent silencing and enhancing for 84 and 172 variable-length exons, respectively.

The microarray detected a splicing change in 213 variable-length exons in KD cells (|ΔIrank|≥1), 147 of which were a result of alternative 5′ splice site use. The accuracy of microarray data was assessed by analysis of seven alternative 5′ splice sites with a 100% validation rate (Figures 5D, 5E, and S9B; Table S4). iCLIP predicted approximately 3% of false positives in either direction among the control exons (|ΔIrank|<0.1; Figure 5B). However, iCLIP predicted a significantly higher number of true positives among the exons that had splicing change in KD cells (*p*<0.05, Fisher's Exact Test; Figure 5B). Among these exons, iCLIP correctly predicted 18 out of 19 exons (95%), of which 8 were enhanced exons (ΔIrank ≥2) and 10 were silenced (ΔIrank ≤−2). For example, a crosslink cluster downstream of the intron-distal alternative 5′ splice site was associated with silencing of the variable portion of exon 11 in CLIP4 pre-mRNA (Figure 5D). In contrast, a crosslink cluster downstream of the intron-proximal alternative 5′ splice site was associated with enhanced inclusion of the variable portion of exon 33 in CHD9 pre-mRNA (Figure 5E).

**Figure 6:**
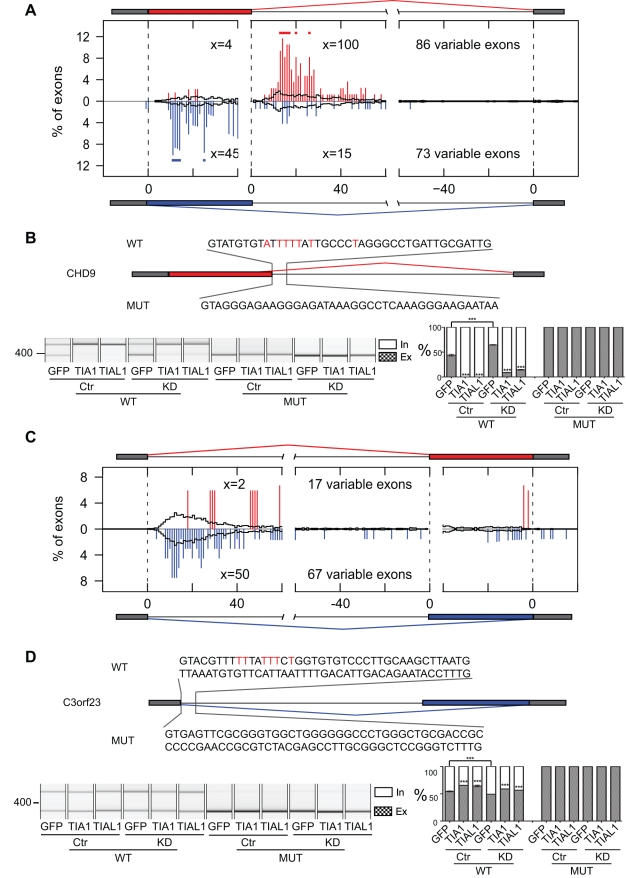
RNA maps of TIA-regulated variable-length exons. RNA maps showing the percentage of variable 5′ splice site exons (A) or variable 3′ splice site exons (C) with clustered TIA1/TIAL1 crosslink sites at exon/intron boundaries of variable exons and flanking constitutive exons, including 20 nt of exonic, 40 nt of variable exonic, and 60 nt of intronic sequence. The number of crosslink events at each region “x” 10–28 nt downstream of exon/intron boundaries and the total number of exons analysed are shown for silenced (ΔIrank ≤1, blue bars), enhanced (ΔIrank ≥1, red bars), and control variable exons (|ΔIrank| ≤0.1, black line). The positions with a significantly higher number of crosslinking events in TIA-regulated RNAs than in control are indicated by red or blue dots above bars (* *p*<0.05, Fisher's Exact Test). Minigene validation of variable 5′ splice site exon in CHD9 pre-mRNA (B) and variable 3′ splice sites exon in C3orf23 pre-mRNA (D) are shown. Depiction and labelling is as described in the legend for Figure 4B.

To further assess the predictive value of iCLIP independently of the microarray, the nine alternative 5′ splice sites with the highest iCLIP cDNA counts at predictive positions were analysed by RT-PCR. RT-PCR detected alternative isoforms for five of these exons, and all of these showed a splicing change in KD cells (Figure S9D). iCLIP correctly predicted the direction of splicing change for all of these exons (Figures 5C). Furthermore, we comprehensively analysed the positions of TIA crosslink events in target RNAs with respect to the observed splicing change (Figure 6A). In agreement with the predictive regulatory logic, there was a significant increase in the number of crosslink events downstream of the enhanced 5′ splice sites, but not at any other positions in the RNA map.

To further verify TIA regulation of an alternative 5′ splice site, we constructed a reporter minigene containing the variable exon 33 and downstream intron and exon from CHD9 pre-mRNA (Figure 6B). The only TIA crosslink cluster present in this region located downstream of the intron-proximal 5′ splice site (Figure 5E). Overexpression of either TIA1 or TIAL1 significantly increased inclusion of the variable portion of the exon, whereas knockdown of the TIA proteins showed the opposite effect, which could again be rescued by TIA overexpression (Figure 6B). Replacing 40 nt of intronic sequence downstream of the intron-proximal 5′ splice site with a sequence from CDC25C pre-mRNA rendered the minigene unresponsive to the changing TIA protein levels (Figure 6B). This confirmed that the TIA crosslink clusters identified the RNA sites that mediated the effect of TIA proteins at the alternative 5′ splice sites.

### TIA Proteins Regulate the Usage of Alternative 3′ Splice Sites by Binding at 5′ Splice Sites

The microarray also detected a splicing change in 84 alternative 3′ splice sites, four of which were analysed by RT-PCR with a 75% (3/4) validation rate (Figure S9C, Table S4). The RNA map of TIA binding showed no enrichment of TIA crosslink events at the regulated alternative 3′ splice sites (Figure 6C). Instead, there was an increase in TIA crosslink event 10–28 nt downstream of the preceding 5′ splice site if TIA silenced inclusion of the variable portion of the exon, as shown for C3orf23 pre-mRNA (Figures 6C and S8B). Although the enrichment did not reach the significance level, these observations indicated that TIA proteins might silence the intron-proximal alternative 3′ splice sites by binding at the preceding 5′ splice site.

In order to test this hypothesis, we constructed a reporter minigene containing variable-length exon 4 and the upstream intron and exon from C3orf23 pre-mRNA (Figure 6D). The only TIA crosslink cluster present located downstream of the 5′ splice site (Figure S8B). Since the intron has a size of more than 6 kb, we fused its first and last 600 nt to form the intron in the minigene. Overexpression of TIA1 or TIAL1 protein significantly decreased the inclusion of the variable part of the exon, whereas TIA knockdown significantly increased the inclusion (Figure 6D). In order to verify functionality of this binding site, the 83 nt downstream of the 5′ splice site were replaced with the corresponding region from constitutive exon 2 of GAPDH pre-mRNA (Figure 6D). This resulted in a loss of TIA regulation. Surprisingly, the mutation abolished inclusion of the variable portion of the exon, possibly due to the enhanced TIA-independent recognition of the GAPDH 5′ splice site. Taken together, the minigene reporter analysis confirmed that TIA binding downstream of the 5′ splice site can affect the usage of distal alternative 3′ splice sites.

## Discussion

The present study predicts the positive and negative effects of TIA proteins on splicing of cassette and variable-length exons based on experimental analysis of in vivo TIA-RNA interactions. Consistent with previous observations, we found strong binding of TIA1 and TIAL1 downstream of 5′ splice sites where it exerts local and distal effects on splicing regulation. In addition, binding of both proteins was also enriched in ncRNAs and 3′ UTR of mRNAs. The latter is consistent with their role in translational repression via binding AU-rich element (ARE) and with the role in stress granules, where they promote the assembly of translation pre-initiation complexes [18],[19],[39],[40]. Further analyses of iCLIP data could therefore provide novel insights into how TIA binding mediates translational inhibition.

As shown in the example of the 3′ UTR of MYC pre-mRNA, TIA1 and TIAL1 crosslinking is quantitatively reproducible at the level of individual nucleotides (Figure 2F). This allowed us to show that TIA1 and TIAL1 have identical RNA binding specificity and that both proteins bind to the same primary RNA sites. This result is interesting from an evolutionary perspective, raising the question of why two proteins with the same specificity have been maintained in the genome. The redundancy between TIA1 and TIAL1 might increase the robustness of gene expression. Moreover, it is likely that the two proteins differ in their interactions with other proteins and in their post-translational modifications, allowing different signalling pathways to regulate activity of the two proteins. Since the relative expression of TIA1 and TIAL1 varies among human tissues [16], this could allow different signalling pathways to modulate expression of TIA-target RNAs in different tissues.

### The Positions of TIA-RNA Interactions Predict the Splicing Effects of TIA1 and TIAL1

TIA crosslink clusters were identified downstream of 5% of alternative and constitutive exons, suggesting that TIA proteins play a widespread role in 5′ splice site recognition. However, in spite of this widespread TIA binding, we were able to use consistent rules to predict the effects of TIA binding on splicing of regulated alternative exons. Assessing TIA crosslinking at the 5′ splice sites of both the alternative and the preceding exon enabled us to distinguish between silenced and enhanced exons. Splice-junction microarray analyses and minigene experiments validated the accuracy of these predictions on a genome-wide scale and at the level of individual regulated exons, supporting the finding that the position of TIA binding on pre-mRNA determines its dual splicing effects.

The ability of iCLIP in predicting the dual TIA splicing effects is particularly noteworthy in the case of variable-length exons, since the distance between alternative splice sites is often very short, as shown in the example of CLIP4 pre-mRNA (Figure 1C). The ability of iCLIP to directly identify crosslink sites and thereby resolve TIA binding at such proximal sites was crucial for the accuracy in separating silenced from enhanced variable-length exons.

### TIA Effects on Variable-Length Exons Allow Evaluation of the Different Models of Splicing Regulation

**Figure 7:**
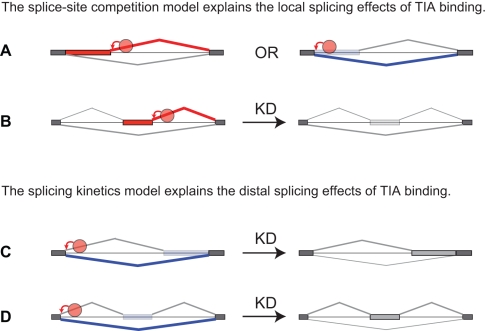
An overview of the models of TIA-dependent splicing regulation. (A) TIA proteins can directly regulate 5′ splice site competition by enhancing either intron-proximal or intron-distal 5′ splice sites. (B) TIA proteins directly regulate alternative cassette exon inclusion by enhancing 5′ splice sites. (C) TIA proteins promote the use of intron-distal alternative 3′ splice sites without directly modulating competition between the alternative 3′ splice sites. By enhancing 5′ splice site recognition, TIA proteins decrease the inclusion of the variable portion of the exon. The splicing kinetics model proposes that the splicing kinetics is affected by 5′ splice site recognition, which then indirectly affects the ability of SR proteins and other factors to define the variable portion of the distal exon. According to this model, the slower splicing kinetics in the absence of TIA increases the time available to these factors to promote inclusion of the variable portion of the exon. (D) Similar to the effect on distal variable exons, a change in splicing kinetics could contribute to the ability of TIA proteins to promote skipping of distal alternative cassette exon by binding at the upstream 5′ splice site.

In the present study, we have evaluated both the local and distal splicing effects of TIA binding at 5′ splice sites. Locally, TIA proteins can regulate alternative 5′ splice sites in a manner consistent with the splice site competition model [41]. TIA proteins enhanced the closest upstream 5′ splice site, leading to a concomitant decrease in usage of the competing 5′ splice site (Figure 7A). Similarly, TIA binding downstream of a cassette exon acts by promoting the usage of its 5′ splice site (Figure 7B). A past study evaluating splicing intermediates showed that in cases of Nova binding downstream of enhanced exons, the downstream intron is removed prior to the upstream one [4]. This result is consistent with a splice site competition model, indicating that Nova and TIA proteins can enhance cassette exons by promoting the splicing pathway that uses the 5′ splice site of the alternative exon, with concomitant decrease in the exon skipping pathway that uses the 5′ splice site of the preceding exon (Figure 7B).

We also found that TIA binding can lead to distal splicing silencing effects. This supports the hypothesis of indirect silencing action, where an RNA-binding protein could cause a distal negative splicing effect by its local enhancing function, initially proposed to explain the same observation in the Nova RNA map [4],[25]. A recent study of PTB-RNA interactions observed an opposite scenario where the local silencing function of PTB causes a distal positive splicing effect [23]. These findings point to the observation that a local splicing function of RNA-binding proteins generally leads to reciprocal distal splicing effects. The study analysing the distal effects of PTB proposed a model where competition between the constitutive and the alternative 5′ splice site was responsible for these distal effects [23].

To gain further insights into the distal regulation of alternative splicing, we analysed the effect of TIA binding on distal alternative splice sites. Surprisingly, we found that TIA proteins regulate usage of alternative 3′ splice sites without binding directly at the 3′ splice sites. This distal effect does not involve a competition between the constitutive and the alternative 5′ splice sites. Instead, TIA proteins regulate the distal alternative 3′ splice sites by modulating recognition of the upstream 5′ splice site (Figure 7C). This result was also supported by the minigene experiment, which showed that a constitutive non-TIA dependent 5′ splice site promotes skipping of the variable portion of the distal exon. This suggests that regulation of a 5′ splice site recognition can affect splicing of downstream alternative 3′ splice sites even if it doesn't compete with another 5′ splice site. This effect could also contribute to the splicing regulation of distal cassette exons (Figure 7D).

### Mechanistic Insights into the TIA-Dependent Regulation of Alternative Splicing

Unlike Nova, FOX2, and PTB proteins [4],[23],[24],[42], which bind at positions close to either 3′ or 5′ splice sites, TIA RNA maps did not identify significant binding at 3′ splice sites or within the alternative exons. Instead, TIA binding was enriched only downstream of the exons, where TIA proteins were reported to recruit U1 snRNP to the 5′ splice sites [9]–[11]. It is clear that the uridine-rich motifs downstream of exons are not the only determinant of TIA binding, since these motifs are present also in the polypyrimidine tracts upstream of exons. It is therefore possible that TIA binds to RNA cooperatively in complex with U1 snRNP, which would ensure TIA binding only downstream of exons.

Interestingly, TIA crosslinking was equally common downstream of constitutive and alternative exons. Past studies found that uridine tracts are among the most enriched motifs downstream of constitutive exons, but the function of TIA binding to these motifs was not validated [15],[43]. We found that TIA binding downstream of constitutive exons often promotes efficient splicing of the corresponding intron. Interestingly, this function is shared with the yeast orthologue Nam8p, suggesting that it represents a primary evolutionary function of the TIA proteins [44]–[46].

We find that TIA proteins can cause a distal splicing effect by regulating recognition of a constitutive 5′ splice site, even if this site does not compete with an alternative 5′ splice site (Figure 7C). Several models of splicing regulation could account for this effect. TIA proteins might change the conformation of the U1 snRNP complex in a way that promotes its pairing with the intron-distal 3′ splice site. Alternatively, binding of TIA proteins could lead to a change in the long-range RNA-RNA interactions, or a change in interactions with other RNA-binding proteins that bind at a distal site. Finally, the result could also be explained in light of the splicing kinetics model. Kinetic parameters of splicing were shown previously to influence the splice site choice [41]. By enhancing U1 snRNP recruitment to the 5′ splice site, TIA proteins might allow the splicing reaction to proceed faster, thereby shortening the time available for definition of the downstream alternative exon. In contrast, the slower splicing kinetics in knockdown cells could allow additional time for trans-acting factors, such as SR proteins, to recognise exonic elements that prevent skipping of the alternative exon (Figure 7C, D) [47]. Such effects of splicing kinetics might be related to the transcriptional effects on splicing, which act partly by changing the ability of SR proteins to define the alternative exons [48]. Taken together, this study of TIA proteins highlights the importance of identifying the positions of protein-RNA interactions with high precision in order to reveal the full complexity of splicing regulation.

## Methods

### iCLIP and iCLAP Methods

HeLa cells were irradiated with UV light. Upon cell lysis, RNA was partially fragmented using RNase I. For iCLIP, TIA1 or TIAL1 were immunoprecipitated with protein G Dynabeads (Invitrogen) conjugated to goat-anti TIA1 (Santa Cruz, C-20) or goat-anti TIAL1 (Santa Cruz, C-18) antibody. For iCLAP, the Strep/His-tagged proteins were affinity purified using Streptavidin and Cobalt beads. RNA was ligated at 3′ ends to an RNA adapter and radioactively labelled on beads. After gel electrophoresis and nitrocellulose membrane transfer, protein-RNA complexes were visualised by autoradiogram. RNA was recovered by proteinase K digestion and reverse transcribed using primers with adapter regions separated by a BamHI restriction site and a barcode region at their 5′ end. cDNA was size-purified, circularised, annealed to an oligonucleotide complementary to the restriction site, and digested with BamHI. Linearised cDNA was then PCR-amplified using primers complementary to the adapter regions and subjected to high-throughput sequencing using Illumina GA2. A more detailed description is available in Text S1.

### Mapping Sequences to the Human Genome

The sequences corresponding to the individual experiment were identified by their defined barcode, the random barcodes were registered, and the barcodes were removed before mapping the sequences to the human genome sequence (version Hg18/NCBI36), allowing one mismatch using Bowtie version 0.10.1 (command line: -a -m 1 -v 1).

### Randomisation of iCLIP Positions

The randomisation was done within co-transcribed regions that were expected to have the same expression levels. For instance, a single gene contains abundant exonic and non-abundant intronic RNA, and intronic RNA contains non-coding RNA genes that are usually highly abundant. We have randomised positions within each individual intron, excluding the non-coding RNA genes. Each non-coding RNA gene was randomised separately. Exons were grouped into one single region for randomisation. However, as evident in Figure 2B, the 3′ UTR contains a higher cDNA enrichment than the coding sequence; therefore, we randomised positions in each UTR region separately from the coding sequence. All annotations were based on the version Hg18/NCBI36 of the human genome sequence. Each cDNA was considered independent when randomising the positions.

### Comparison of Crosslinking Nucleotide Positions

Only crosslink positions (but not the cDNA count) were compared between the different datasets. Crosslink positions in the first dataset define the position of 0 when analysing the positions in the second dataset.

### Analysis of Enriched Pentamers

For sequence analysis of iCLIP crosslink sites, the position of the crosslinking site was extended 10 nt in both directions. The *z* score for pentamer enrichment at the 21 nt region surrounding the crosslink sites was then calculated relative to randomised genomic positions. A more detailed description is available in Text S1.

### Identification of Significant iCLIP Crosslink Sites

This followed the same statistical approach as the analysis of CLIP sequence clusters [42] with a few modifications as described in [28]. Rather than combining crosslink sites into larger clusters, the crosslink sites within the clusters were kept individually in order to preserve the nucleotide resolution of the data. A more detailed description is available in Text S1.

### siRNA Knockdown

Three different oligonucleotides were used together with a scrambled control (Invitrogen, 12935-112). The following siRNA oligonucleotides were used:

siTIA1-1: 5′-GCAAGUUCCUGCAUAUGGAAUGUAU-3′

siTIA1-2: 5′-AGAAUAUCAGAUGCCCGAUGGUAA-3′

siTIA1-3: 5′-GGCAACAGGAAAGUCUAAGGGAUAU-3′

siTIAL1-1: 5′-CGGAUAUGGUUGGCAAGUUACCAA-3′

siTIAL1-2: 5′-CCGAACCAAUUGGGCCACUCGUAAA-3′

siTIAL1-3: 5′-GCGUCUGGGUUAACAGAUCAGCUUA-3′

5 nM of each siRNA (siTIA1 and siTIAL1) were transfected using Lipofectamine iMax (Invitrogen) according to manufacturer's instructions. Cells were transfected again 2 d after the first transfection and were harvested 2 d after the second transfection for protein and RNA analyses.

### Western Blot

The protein concentration was determined using Lowry's Assay (Bio-RAD). Goat anti-TIA1 antibody (1∶1000) (Santa Cruz, C-20) and anti-TIAL1 antibody (1∶1000) (Santa Cruz, C-18) were used to detect TIA1 and TIAL1 protein. Rabbit anti-GAPDH (1∶5000) (Cell signalling) was used for loading control. For overexpression, mouse anti-Strep tag antibody (1∶1000) (Qiagen) was used to detect the tagged protein. Donkey anti-goat HRP, goat anti-rabbit HRP, and goat anti-mouse HRP (Invitrogen) were used as secondary antibodies. The membrane was visualised using ECL kit (Amersham).

### Splice-Junction Microarray

A total of six samples were used, three from the siRNA control and three from KD3. The high-resolution splice-junction microarrays were produced by Affymetrix, monitoring 260,488 exon-exon junctions (each with eight probes) and 315,137 exons (each with 10 probes). cDNA samples were prepared using the GeneChip WT cDNA Synthesis and Amplification Kit (Affymetrix). Analysis of microarray data was done using version 3 of ASPIRE (Analysis of SPlicing Isoform Reciprocity). ASPIRE predicts splicing changes from reciprocal sets of microarray probes that recognise either inclusion or skipping of an alternative exon. In version 3 the background detection levels are experimentally determined for each probe, allowing background subtraction in a probe-specific manner [28]. Description of RT-PCR validation is available in Text S1.

### iCLIP RNA Maps

RNA maps were produced by assessing the positioning of clustered crosslink sites at the exon/intron boundaries of alternative and flanking exons. For each exon, the positions between 20 nt of exonic and 60 nt of intronic sequence for each of the splice sites were analysed. When introns or exons were shorter than two times the length of the analysed area, analysis was restricted to region up the middle of the intron and exon. To draw the RNA map, the percentage of exons containing a crosslink site at the corresponding position is drawn.

### Minigene Construction and Transfection

The alternative exons with the flanking constitutive exons were amplified by PCR with genomic DNA from HeLa cells and cloned into pcDNA3 vectors. The 40 nt downstream of the exon-intron boundaries where TIA binding site are present were mutated to sequences with no observed TIA binding sites. In the case of the enhanced cassette exon (OGT1) and the alternative 5′ splice sites (CHD9), the 40 nt were replaced with sequences downstream of a silenced cassette exon (CDC25C). In the case of the alternative 3′ splice sites (C3orf23), the 83 nt were replaced with sequences downstream of GAPDH exon 2. The intron of C3orf23 was too long, so only 600 nt from the exon-intron boundaries on either side were cloned. The constructs were transfected together with GFP, TIA1, or TIAL1-pcDNA3/Step-His vectors using polyfect (Qiagen). The siRNA for TIA1 (KD3) and negative control were transfected at the same time using Lipofectamine iMax (Invitrogen). The cells were collected 2 d later and the splicing effects were assessed by RT-PCR using a T7 forward primer (5′-TAATACGACTCACTATAGGG-3′) and gene-specific reverse primers (Table S4).

## Supporting Information

Figure S1 **iCLIP and iCLAP of TIA1 and TIAL1.** (A) Western blot for TIA1 and TIAL1 in HeLa, TIA1 or TIAL1 overexpressed, and TIA1/TIAL1 double KD cells. GAPDH was used as loading control. (B) Western blot with either antibody upon immunoprecipitation using the same antibody. Protein G beads were used for immunoprecipitation. The strong signal at ±130 kDa represents immunoglobulin cross-reactivity. A weak signal for native TIA1 and TIAL1 is seen at ±50 kDa, and the overexpressed proteins migrate at only slightly higher molecular weight. (C) Autoradiogram of ^32^P-γ-ATP labelled RNA in complex with TIA1 or TIAL1 in iCLIP. The low RNase I results in a shift of the complex. (D) The final PCR gel for each protein before being submitted to sequencing on the Illumina GA2 system. (E) iCLAP purification of overexpressed TIA1 and TIAL1 with Strep and His tag. The proteins were first purified using Strep beads and then purified with Cobalt beads. (F) iCLAP autoradiogram for TIA1 and TIAL1. Vector-transfected cells and no UV-crosslinking samples were used as controls.
(1.51 MB PDF)

Figure S2 **A global view of replicate iCLIP and iCLAP experiments for TIA1 and TIAL1.** The three individual replicates of iCLIP for TIA1 or TIAL1 together with iCLAP for either protein are shown in BedGraph format in the UCSC hg18 Genome Browser. cDNA counts at crosslink sites on the sense (purple) or anti-sense strand (orange) of the chromosome 20 are shown. The cDNA counts are shown on the left of the BedGraphs.
(0.27 MB PDF)

Figure S3 **Reproducibility of replicate iCLIP and iCLAP experiments for TIA1 and TIAL1.** (A) Fold-enrichment of pentamers in the 21 nt sequence surrounding crosslink sites (−10 nt to +10 nt) are shown for TIA1 and TIAL1 iCLIP. (B) Pentamer *z* scores at the 21 nt sequence surrounding crosslink sites (−10 nt to +10 nt) are shown for TIA1 and TIAL1 iCLAP. The sequences for the two most enriched pentamers and the Pearson correlation coefficient (*r*) are shown. (C,D) Reproducibility of sequence composition at crosslink nucleotides. Frequencies of pentanucleotides overlapping with crosslink nucleotides are shown for the three replicate experiments for TIA1 (C) or TIAL1 (D). (E) Weblogo showing base frequencies of crosslink nucleotides and 20 nt of surrounding genomic sequence. Positions 0 and 1 correspond to crosslink nucleotide and first position of cDNA sequence, respectively. (F) Reproducibility analysis comparing the positions of clustered crosslink sites of TIA1 and TIAL1. Black bars show the number of crosslink nucleotides from TIA1 iCLIP that are reproduced in TIAL1 iCLIP with a given offset. An offset of 0 nt indicates the number of crosslink nucleotides from TIA1 iCLIP that were reproduced by a crosslink nucleotide at exactly the same position in TIAL1 iCLIP. Negative or positive offset values indicate whether the reproducing TIAL1 crosslink position is located upstream or downstream of the TIA1 crosslink nucleotide, respectively. The orange curve depicts results of the same analysis upon randomisation of TIA1 crosslink nucleotide positions. (G–I) Contour plots comparing TIA1 and TIAL1 cDNA enrichment in ncRNAs (G), 3′ UTRs (H), and introns (I). The cDNA enrichment was calculated by dividing the density in each RNA region by the whole-genome cDNA density. The legend shows the minimal density of RNAs in an area of the plot corresponding to the colour of each contour.
(0.59 MB PDF)

Figure S4 **TIA1 and TIAL1 crosslink to different parts of chromosomes.** (A) Global view of chromosome 6 with TIA1 and TIAL1 iCLIP crosslink sites. The chromosome is shown at the top. The genes are shown below the tracks. The zoom-in view shows the MAPK14 gene. This gene has two mutually exclusive exons, and splicing change was detected by the microarray. The regions with enriched iCLIP cDNAs were zoomed in further down to nucleotide resolution. (B) Global view of the same chromosome. This time, the region with very high numbers of cDNAs was zoomed in. The crosslink sites map to the antisense strand, and three major peaks were further zoomed in. The first one maps to the 3′ UTR of a histone gene (1), whereas the other two map to non-coding tRNAs (2, 3).
(0.71 MB PDF)

Figure S5 **The location of TIA1 and TIAL1 crosslink sites in previously described pre-mRNAs.** (A) TIA1 and TIAL1 crosslink sites in FAS pre-mRNA. In the mid panel, exons 5, 6, and 7 are shown, with exon 6 being alternatively spliced. The arrows above the bar graphs show the previously identified TIA binding sites. (B) TIA1 and TIAL1 crosslink sites in MYC pre-mRNA. Most of the sites were concentrated at the 3′ UTR. Upper and lower panels depict enlarged regions showing nucleotide resolution of iCLIP crosslink sites.
(0.29 MB PDF)

Figure S6 **TIA1/TIAL1 siRNA knock-down in HeLa cells.** Western blot for TIA1/TIAL1 KD samples. Either TIA1 or TIAL1 was detected in the upper panels and GAPDH in the bottom panel as loading control.
(0.19 MB PDF)

Figure S7 **TIA1/TIAL1 regulate intron retention.** (A) iCLIP crosslink sites in the silenced intron in PPIA pre-mRNA. The exon is shown by the rectangle, and the alternative intron by the arrowed line. The area surrounding the 5′ splice site is shown at a greater resolution below. (B) 18 intron retention events detected by the microarray were analysed by real-time PCR in control and TIA1/TIAL1 KD samples (prepared using the third siRNA oligonucleotide).
(0.33 MB PDF)

Figure S8 **TIA binding causes distal splicing effects.** (A) UCSC hg18 Genome Browser views of iCLIP crosslink sites around the two mutually exclusive exons of FYN pre-mRNA. The areas surrounding both 5′ splice sites are shown at a higher resolution. Capillary electrophoresis of RT-PCR from KD samples and its quantification are shown below. (B) UCSC Genome Browser views of the alternative 3′ splice site in C3orf23 pre-mRNA. The areas surrounding the 5′ splice site of the preceding exon and the alternative 3′ splice sites are shown at a higher resolution. Capillary electrophoresis of RT-PCR from KD samples and its quantification are shown below (* *p*<0.05, *** *p*<0.001, one-way ANOVA).
(0.43 MB PDF)

Figure S9 **RT-PCR validation of splicing events detected by the microarray using QIAxcel.** (A) Cassette exons events validated by QIAxcel and their quantification are shown next to the pictures. (B) Validated alternative 5′ splice sites regulated by the TIA proteins. (C) Validated alternative 3′ splice sites regulated by the TIA proteins. (D) Validated alternative 5′ splice sites predicted by iCLIP. * *p*<0.05, ** *p*<0.01, *** *p*<0.001, one-way ANOVA. (A–D) Depiction and labelling as in Figure 3D–E. (E) The percentage of change in exon inclusion (ΔI) detected by microarray and RT-PCR are plotted against each other.
(1.10 MB PDF)

Table S1 **Information used to map the sequencing results to genome.**
(0.04 MB PDF)

Table S2 **Mapping information for iCLIP and iCLAP data.**
(0.03 MB PDF)

Table S3 Enrichment of pentamers surrounding TIA1 and TIAL1 iCLIP and iCLAP crosslink sites.
(0.02 MB PDF)

Table S4 **RT-PCR primers for validation of splicing event predicted by the microarray.**
(0.08 MB PDF)

Table S5 **qPCR primers for validation of intron retention event.**
(0.06 MB PDF)

Text S1 **Supplementary methods.** A detailed explanation of the iCLIP, iCLAP, *z* score analysis, identification of significant iCLIP crosslink sites, and RT-PCR analysis is provided.
(0.04 MB DOC)

## Abbreviations

3′ UTR: 3′ untranslated region
ARE: AU-rich element
iCLAP: individual-nucleotide resolution UV-crosslinking and affinity purification
iCLIP: individual-nucleotide resolution UV-crosslinking and immunoprecipitation
KD: knockdown
ncRNA: non-coding RNA
nt: nucleotide
RRMs: RNA recognition motifs
snRNP: small nuclear ribonucleoprotein particles
TIA1: T-cell intracellular antigen 1
TIAL1: TIA1-like 1 or TIA1-related
