# Supplementary figures and images for: iCLIP Predicts the Dual Splicing Effects of TIA-RNA Interactions
Source: PLoS Biol. 2010 Oct 26;8(10):e1000530. doi: 10.1371/journal.pbio.1000530 (PMC2964331; doi:10.1371/journal.pbio.1000530)

**A**

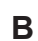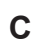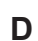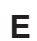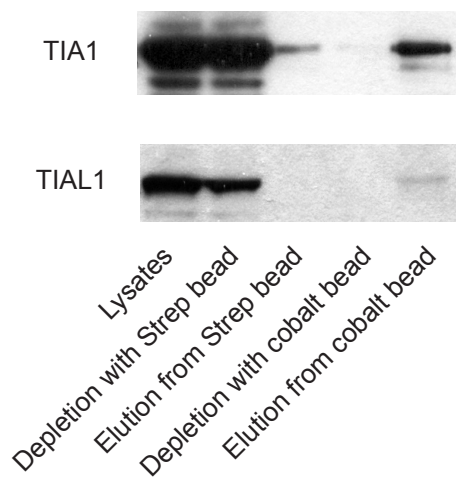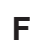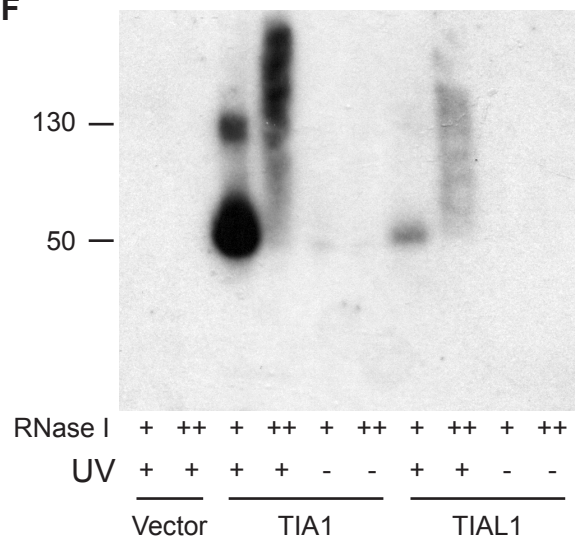

Supplement: Figure S1 — iCLIP and iCLAP of TIA1 and TIAL1. (A) Western blot for TIA1 and TIAL1 in HeLa, TIA1 or TIAL1 overexpressed, and TIA1/TIAL1 double KD cells. GAPDH was used as loading control. (B) Western blot with either antibody upon immunoprecipitation using the same antibody. Protein G beads were used for immunoprecipitation. The strong signal at ±130 kDa represents immunoglobulin cross-reactivity. A weak signal for native TIA1 and TIAL1 is seen at ±50 kDa, and the overexpressed proteins migrate at only slightly higher molecular weight. (C) Autoradiogram of 32P-γ-ATP labelled RNA in complex with TIA1 or TIAL1 in iCLIP. The low RNase I results in a shift of the complex. (D) The final PCR gel for each protein before being submitted to sequencing on the Illumina GA2 system. (E) iCLAP purification of overexpressed TIA1 and TIAL1 with Strep and His tag. The proteins were first purified using Strep beads and then purified with Cobalt beads. (F) iCLAP autoradiogram for TIA1 and TIAL1. Vector-transfected cells and no UV-crosslinking samples were used as controls. (1.51 MB PDF) [file pbio.1000530.s001.pdf]

Figure S2

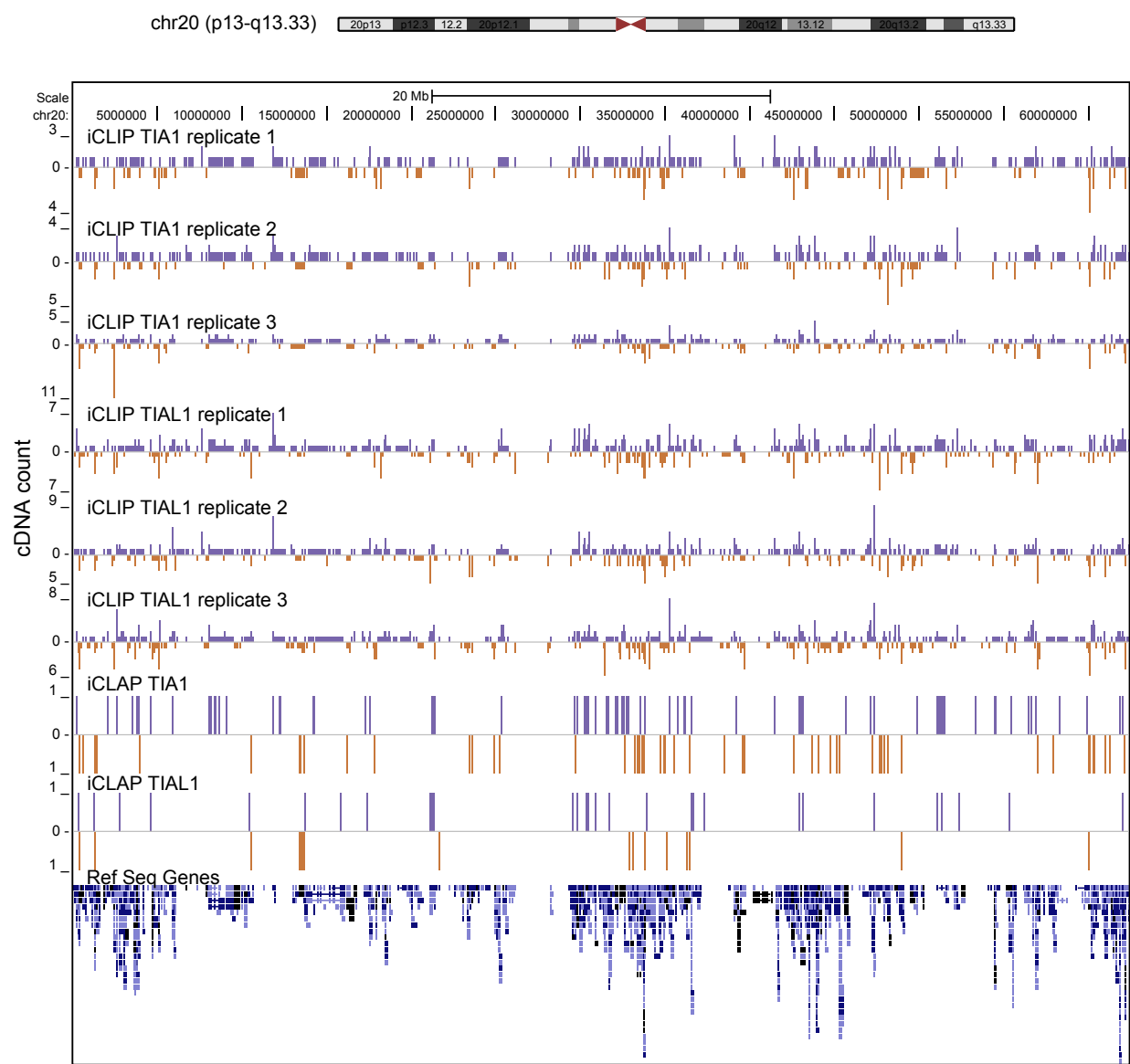

Supplement: Figure S2 — A global view of replicate iCLIP and iCLAP experiments for TIA1 and TIAL1. The three individual replicates of iCLIP for TIA1 or TIAL1 together with iCLAP for either protein are shown in BedGraph format in the UCSC hg18 Genome Browser. cDNA counts at crosslink sites on the sense (purple) or anti-sense strand (orange) of the chromosome 20 are shown. The cDNA counts are shown on the left of the BedGraphs. (0.27 MB PDF) [file pbio.1000530.s002.pdf]

**A**

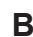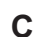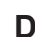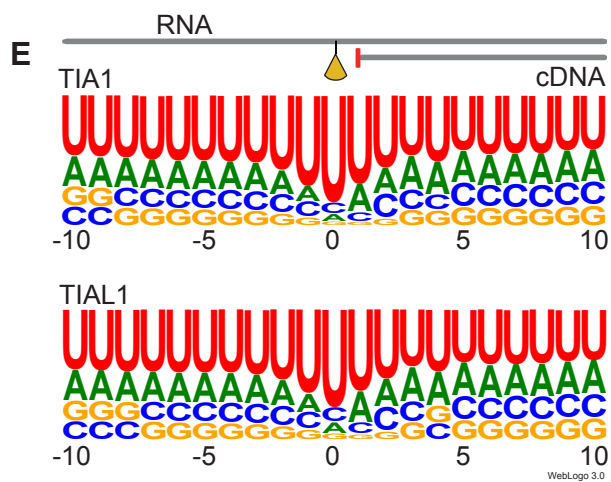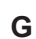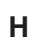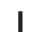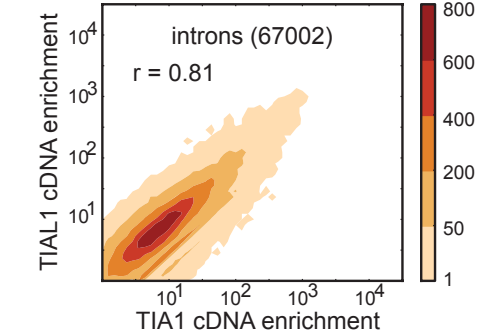

Supplement: Figure S3 — Reproducibility of replicate iCLIP and iCLAP experiments for TIA1 and TIAL1. (A) Fold-enrichment of pentamers in the 21 nt sequence surrounding crosslink sites (−10 nt to +10 nt) are shown for TIA1 and TIAL1 iCLIP. (B) Pentamer z scores at the 21 nt sequence surrounding crosslink sites (−10 nt to +10 nt) are shown for TIA1 and TIAL1 iCLAP. The sequences for the two most enriched pentamers and the Pearson correlation coefficient (r) are shown. (C,D) Reproducibility of sequence composition at crosslink nucleotides. Frequencies of pentanucleotides overlapping with crosslink nucleotides are shown for the three replicate experiments for TIA1 (C) or TIAL1 (D). (E) Weblogo showing base frequencies of crosslink nucleotides and 20 nt of surrounding genomic sequence. Positions 0 and 1 correspond to crosslink nucleotide and first position of cDNA sequence, respectively. (F) Reproducibility analysis comparing the positions of clustered crosslink sites of TIA1 and TIAL1. Black bars show the number of crosslink nucleotides from TIA1 iCLIP that are reproduced in TIAL1 iCLIP with a given offset. An offset of 0 nt indicates the number of crosslink nucleotides from TIA1 iCLIP that were reproduced by a crosslink nucleotide at exactly the same position in TIAL1 iCLIP. Negative or positive offset values indicate whether the reproducing TIAL1 crosslink position is located upstream or downstream of the TIA1 crosslink nucleotide, respectively. The orange curve depicts results of the same analysis upon randomisation of TIA1 crosslink nucleotide positions. (G–I) Contour plots comparing TIA1 and TIAL1 cDNA enrichment in ncRNAs (G), 3′ UTRs (H), and introns (I). The cDNA enrichment was calculated by dividing the density in each RNA region by the whole-genome cDNA density. The legend shows the minimal density of RNAs in an area of the plot corresponding to the colour of each contour. (0.59 MB PDF) [file pbio.1000530.s003.pdf]

Figure S4

A

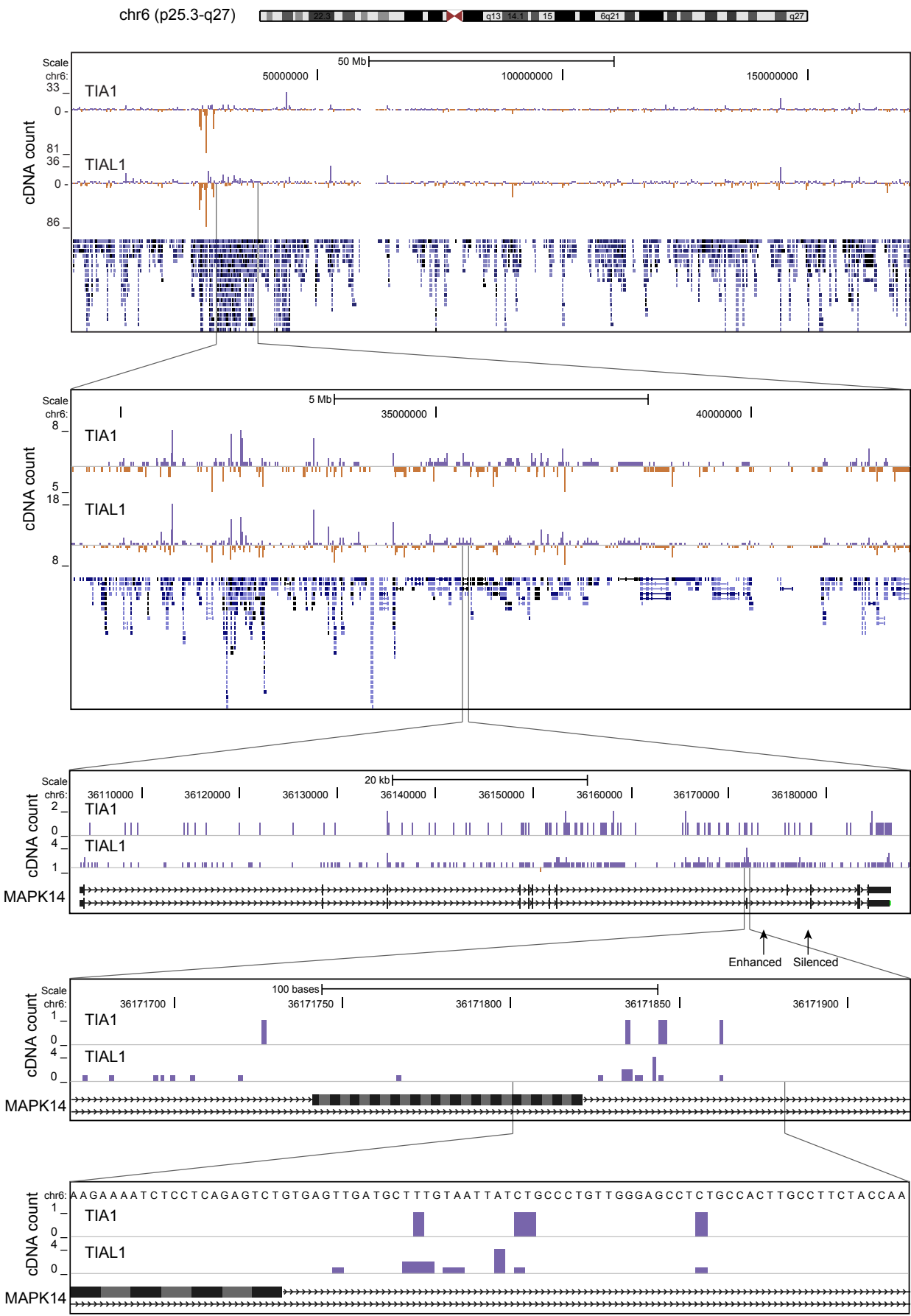

Figure S4

B

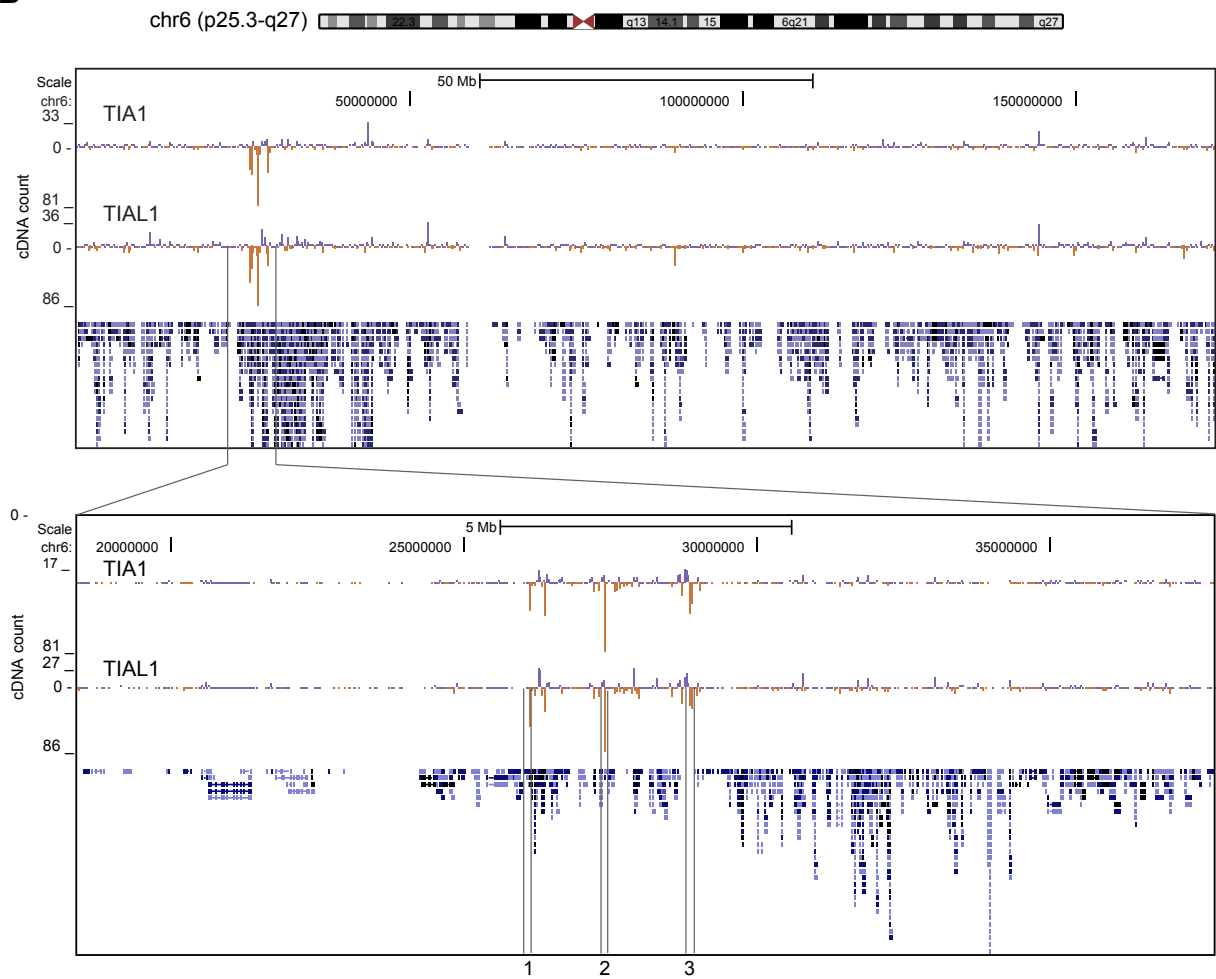

1

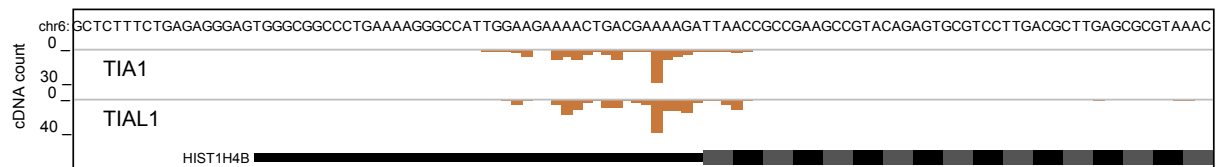

2

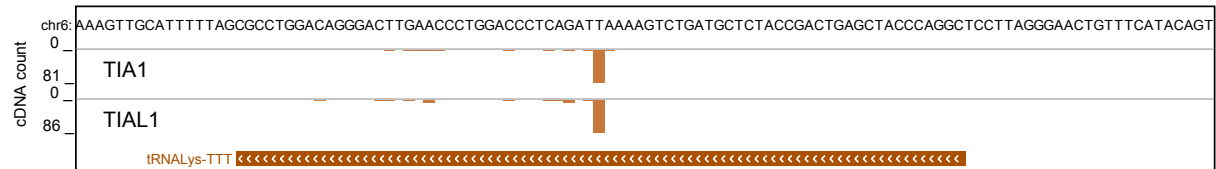

3

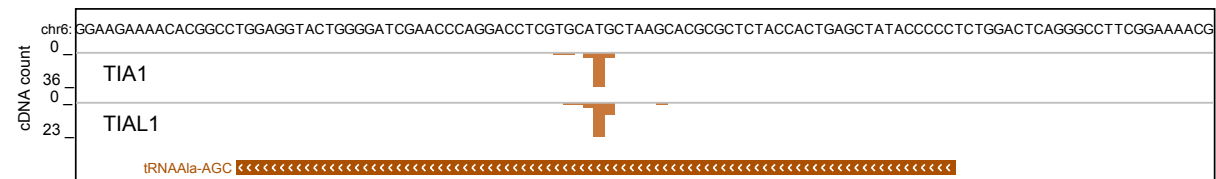

Supplement: Figure S4 — TIA1 and TIAL1 crosslink to different parts of chromosomes. (A) Global view of chromosome 6 with TIA1 and TIAL1 iCLIP crosslink sites. The chromosome is shown at the top. The genes are shown below the tracks. The zoom-in view shows the MAPK14 gene. This gene has two mutually exclusive exons, and splicing change was detected by the microarray. The regions with enriched iCLIP cDNAs were zoomed in further down to nucleotide resolution. (B) Global view of the same chromosome. This time, the region with very high numbers of cDNAs was zoomed in. The crosslink sites map to the antisense strand, and three major peaks were further zoomed in. The first one maps to the 3′ UTR of a histone gene (1), whereas the other two map to non-coding tRNAs (2, 3). (0.71 MB PDF) [file pbio.1000530.s004.pdf]

Figure S5

A

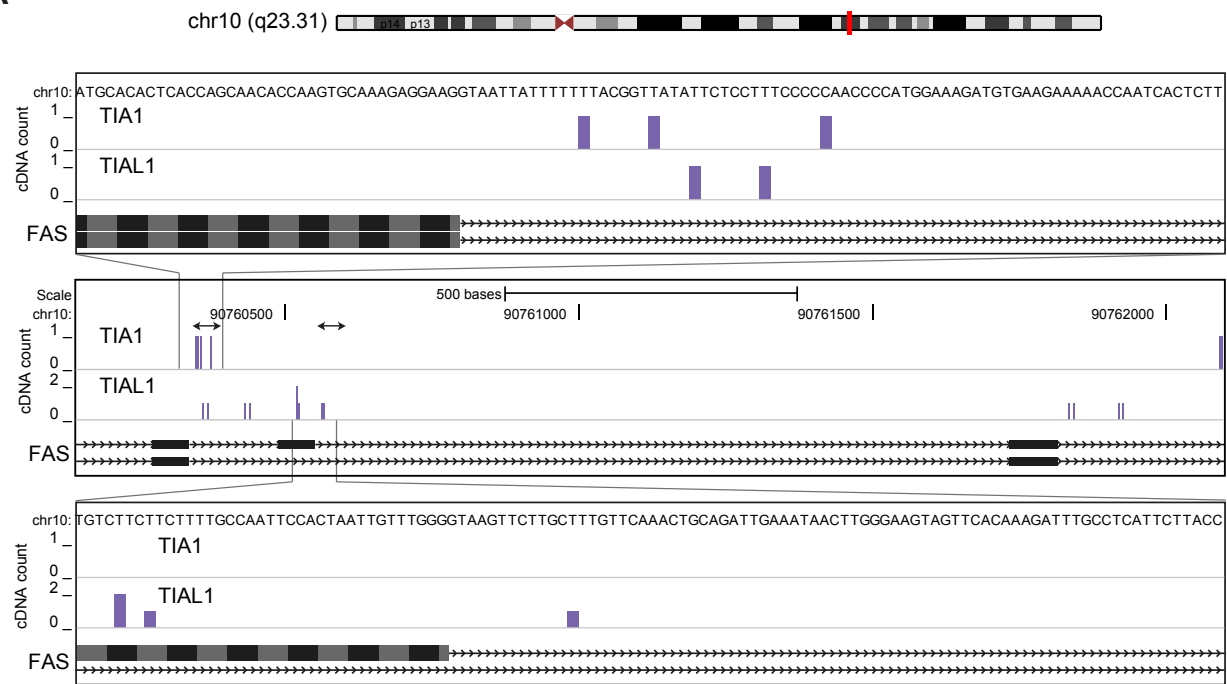

B

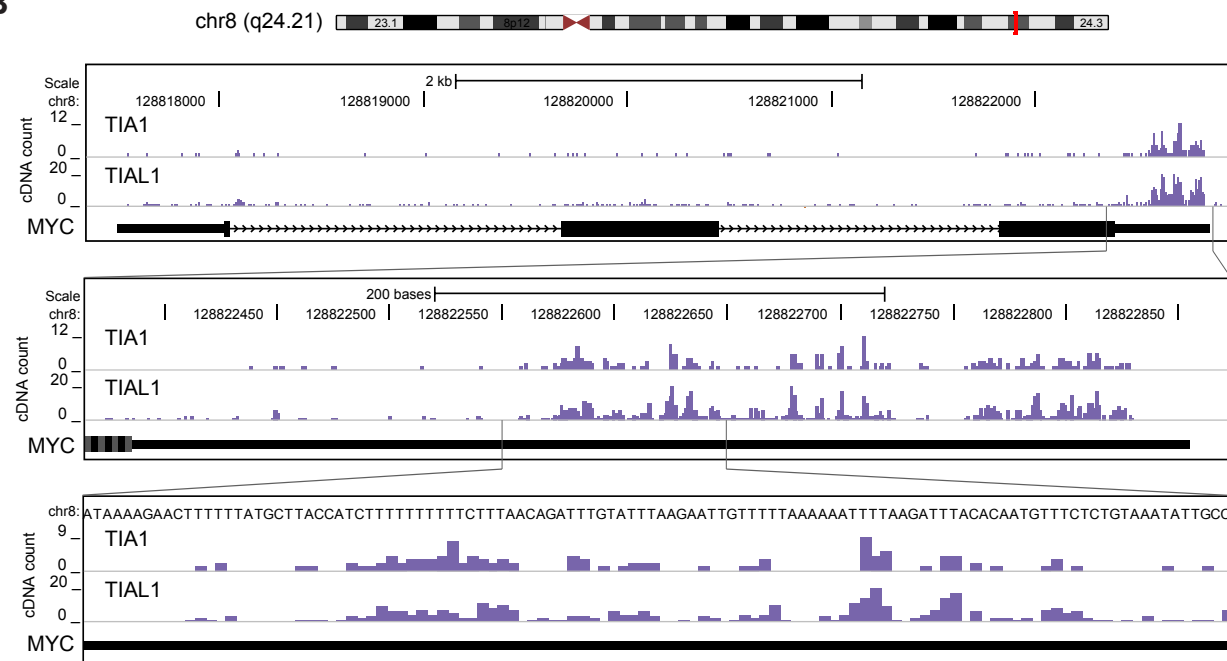

Supplement: Figure S5 — The location of TIA1 and TIAL1 crosslink sites in previously described pre-mRNAs. (A) TIA1 and TIAL1 crosslink sites in FAS pre-mRNA. In the mid panel, exons 5, 6, and 7 are shown, with exon 6 being alternatively spliced. The arrows above the bar graphs show the previously identified TIA binding sites. (B) TIA1 and TIAL1 crosslink sites in MYC pre-mRNA. Most of the sites were concentrated at the 3′ UTR. Upper and lower panels depict enlarged regions showing nucleotide resolution of iCLIP crosslink sites. (0.29 MB PDF) [file pbio.1000530.s005.pdf]

Figure S6

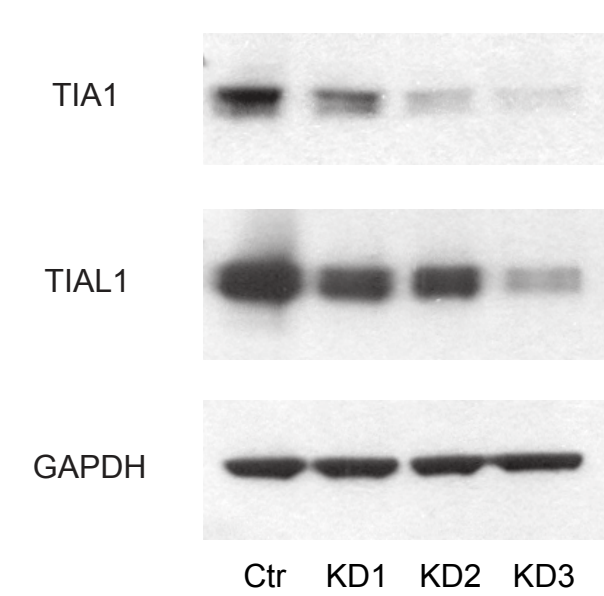

Supplement: Figure S6 — TIA1/TIAL1 siRNA knock-down in HeLa cells. Western blot for TIA1/TIAL1 KD samples. Either TIA1 or TIAL1 was detected in the upper panels and GAPDH in the bottom panel as loading control. (0.19 MB PDF) [file pbio.1000530.s006.pdf]

Supplementary Figure 7

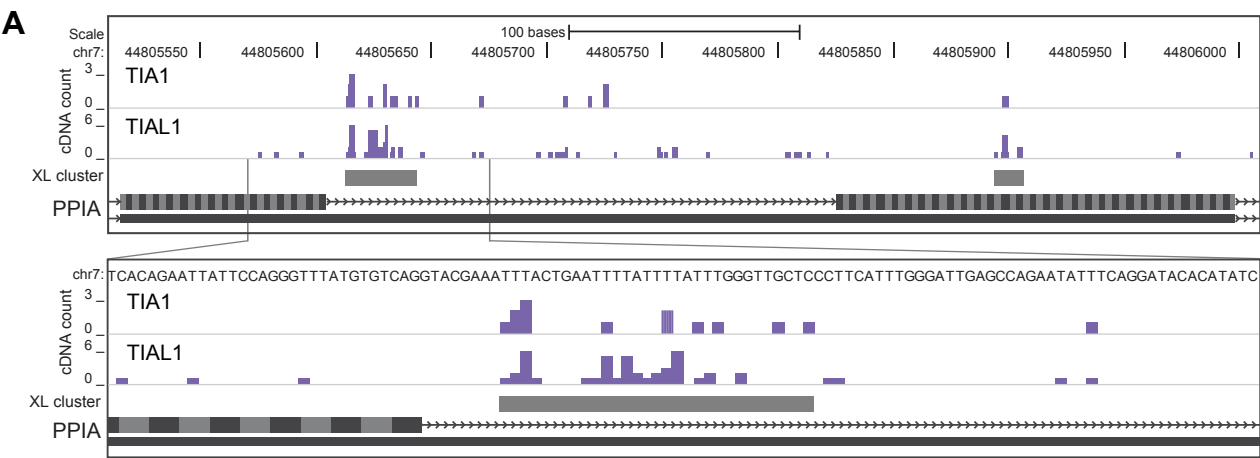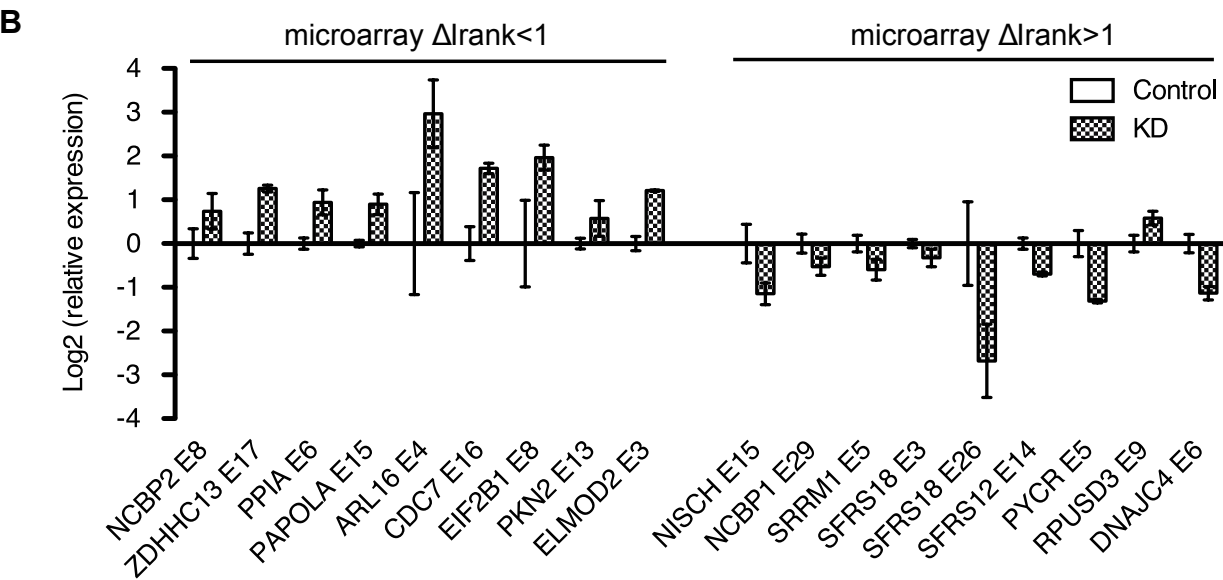

Supplement: Figure S7 — TIA1/TIAL1 regulate intron retention. (A) iCLIP crosslink sites in the silenced intron in PPIA pre-mRNA. The exon is shown by the rectangle, and the alternative intron by the arrowed line. The area surrounding the 5′ splice site is shown at a greater resolution below. (B) 18 intron retention events detected by the microarray were analysed by real-time PCR in control and TIA1/TIAL1 KD samples (prepared using the third siRNA oligonucleotide). (0.33 MB PDF) [file pbio.1000530.s007.pdf]

Figure S8

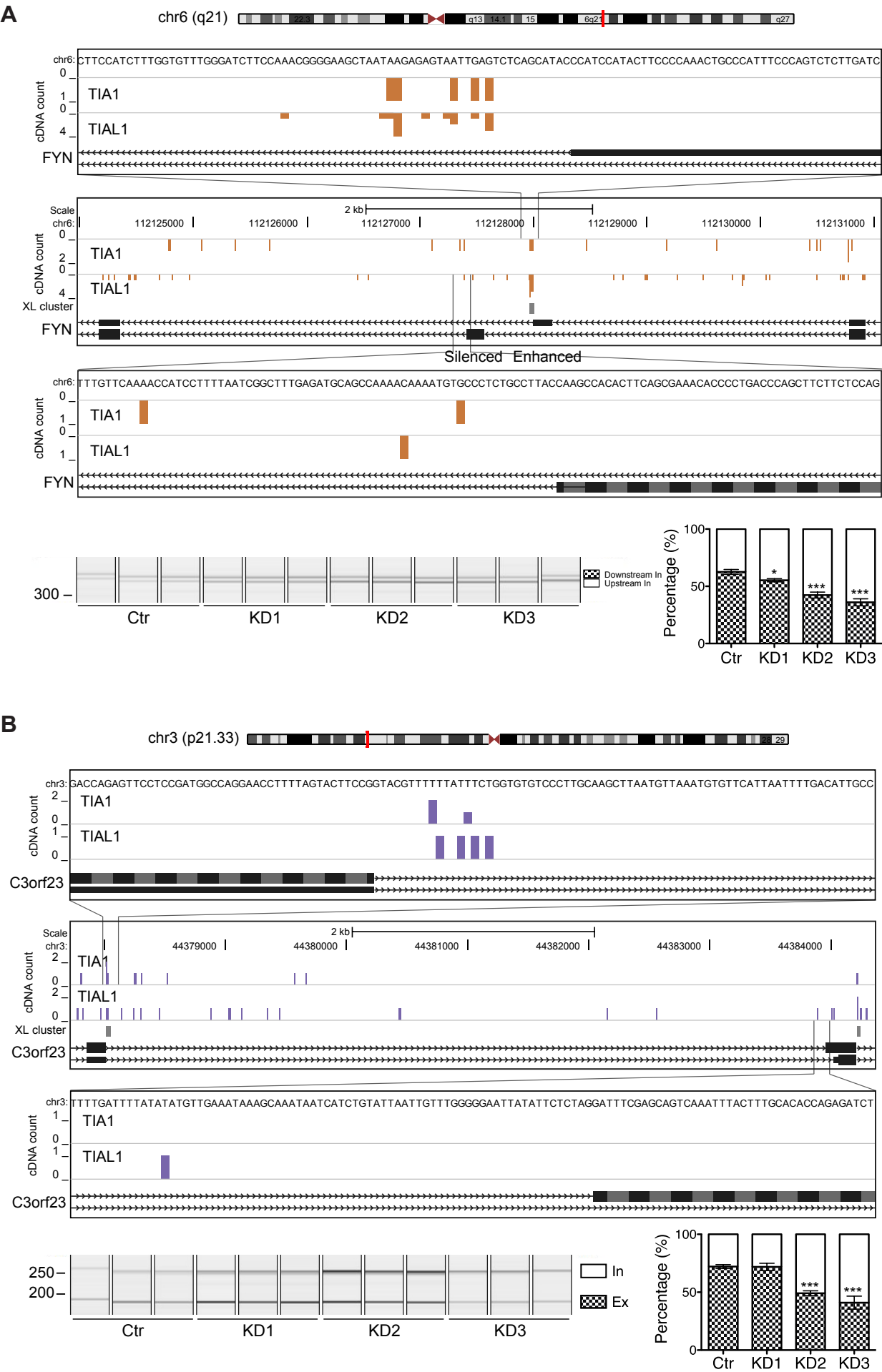

Supplement: Figure S8 — TIA binding causes distal splicing effects. (A) UCSC hg18 Genome Browser views of iCLIP crosslink sites around the two mutually exclusive exons of FYN pre-mRNA. The areas surrounding both 5′ splice sites are shown at a higher resolution. Capillary electrophoresis of RT-PCR from KD samples and its quantification are shown below. (B) UCSC Genome Browser views of the alternative 3′ splice site in C3orf23 pre-mRNA. The areas surrounding the 5′ splice site of the preceding exon and the alternative 3′ splice sites are shown at a higher resolution. Capillary electrophoresis of RT-PCR from KD samples and its quantification are shown below (* p<0.05, *** p<0.001, one-way ANOVA). (0.43 MB PDF) [file pbio.1000530.s008.pdf]
