# Supplementary material for: iCLIP Predicts the Dual Splicing Effects of TIA-RNA Interactions
Source: PLoS Biol. 2010 Oct 26;8(10):e1000530. doi: 10.1371/journal.pbio.1000530 (PMC2964331; doi:10.1371/journal.pbio.1000530)

Supplementary Figure 9

A Cassette exons

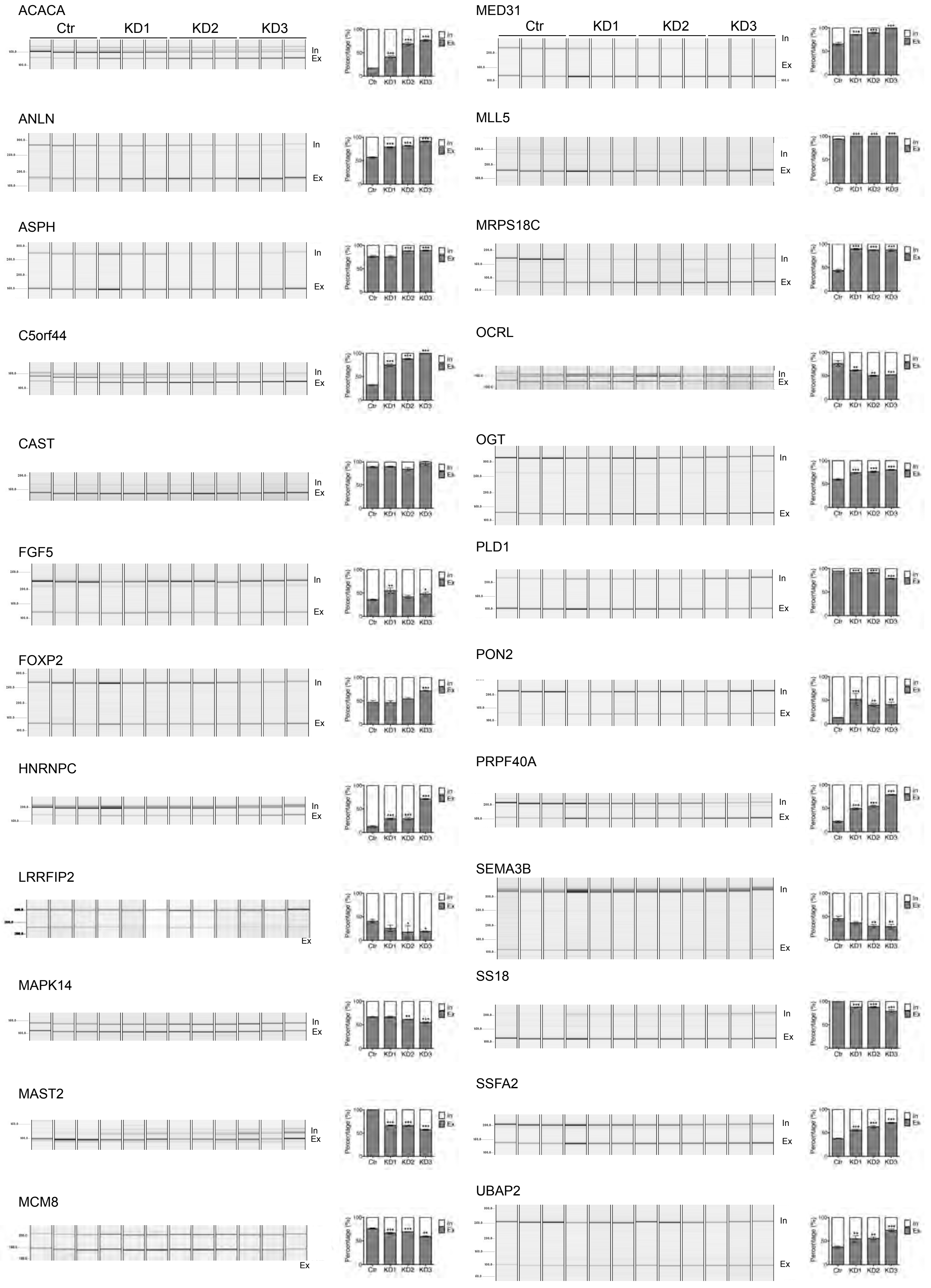

Supplementary Figure 9

B Alternative 5' splice sites

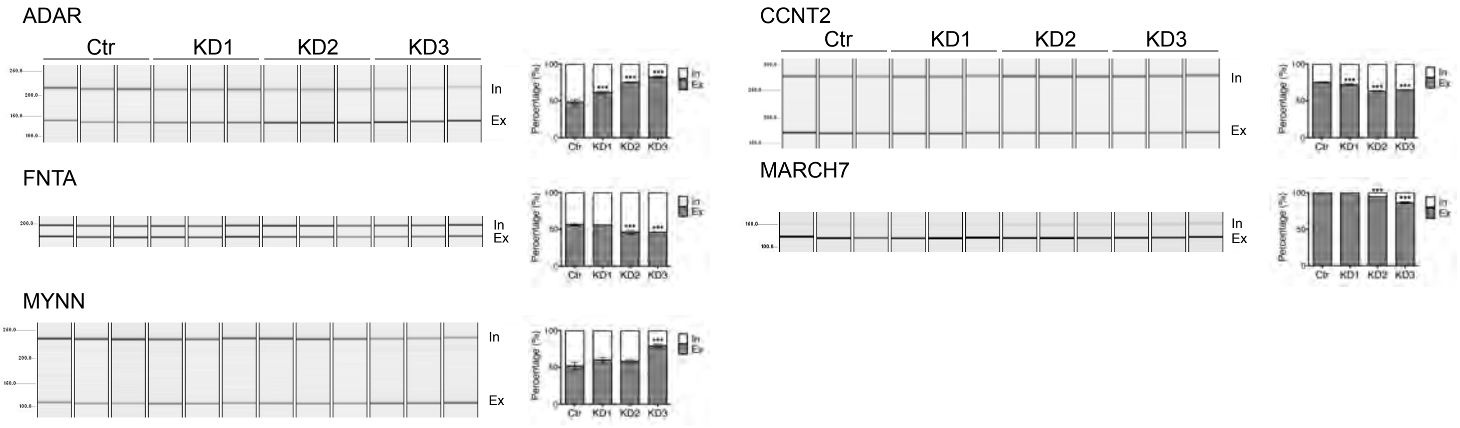

C Alternative 3' splice sites

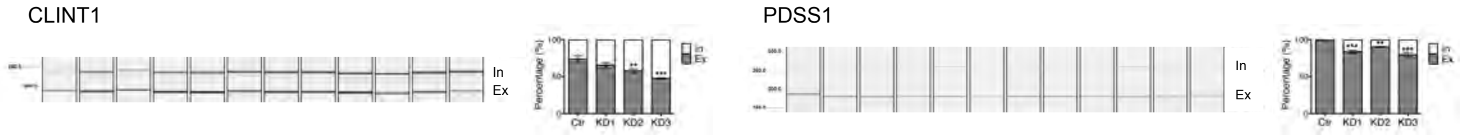

D Predicted alternative 5' splice sites

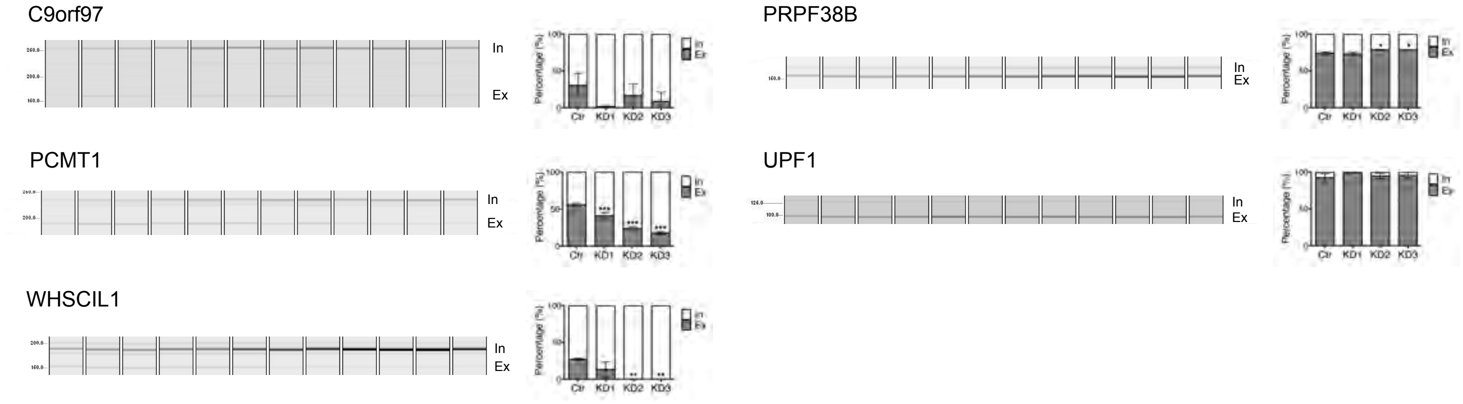

E

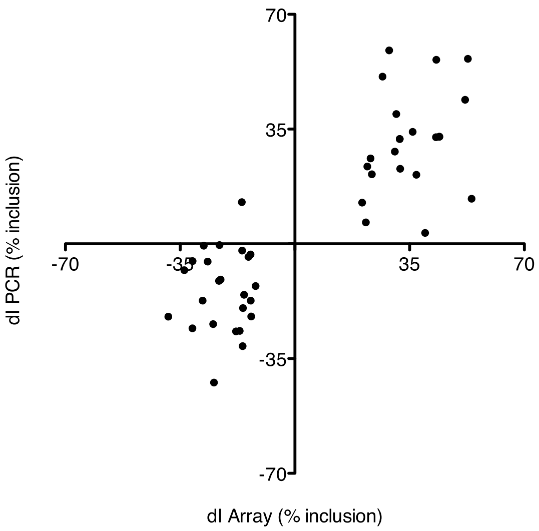

Supplement: Figure S9 — RT-PCR validation of splicing events detected by the microarray using QIAxcel. (A) Cassette exons events validated by QIAxcel and their quantification are shown next to the pictures. (B) Validated alternative 5′ splice sites regulated by the TIA proteins. (C) Validated alternative 3′ splice sites regulated by the TIA proteins. (D) Validated alternative 5′ splice sites predicted by iCLIP. * p<0.05, ** p<0.01, *** p<0.001, one-way ANOVA. (A–D) Depiction and labelling as in Figure 3D–E. (E) The percentage of change in exon inclusion (ΔI) detected by microarray and RT-PCR are plotted against each other. (1.10 MB PDF) [file pbio.1000530.s009.pdf]
