# Supplementary material for: iCLIP Predicts the Dual Splicing Effects of TIA-RNA Interactions
Source: PLoS Biol. 2010 Oct 26;8(10):e1000530. doi: 10.1371/journal.pbio.1000530 (PMC2964331; doi:10.1371/journal.pbio.1000530)
Supplement: Table S1 — Information used to map the sequencing results to genome. (0.04 MB PDF) [file pbio.1000530.s010.pdf]

Table S1. Information used to map the sequencing result to genome.

Replicate	Protein	Method	Species	5' define barcode	3'	5' random barcode	3' barcode	File	Condition
1	TIA1	iCLIP	hu	TG_0	single	NNN_2:4	TG	LUd3.090715.42L2VAAXX.s_7.fq	60-90 kDa cut
1	TIA1	iCLIP	hu	GA_0	single	NNN_2:4	GA	LUd3.090715.42L2VAAXX.s_7.fq	90-130 kDa cut
2	TIA1	iCLIP	hu	TC_0	single	NNN_2:4	AT	LUd4.090715.42L7HAAXX.s_1.fq	60-90 kDa cut
2	TIA1	iCLIP	hu	CA_0	single	NNN_2:4	CC	LUd4.090715.42L7HAAXX.s_1.fq	60-90 kDa cut
2	TIA1	iCLIP	hu	AG_0	single	NNN_2:4	CC	LUd4.090715.42L7HAAXX.s_1.fq	90-130 kDa cut
3	TIA1	iCLIP	hu	GTT_0	single	NNNN_3:6	TG	LUd15.090831.42KYHAAXX.s_6.fq	60-90 kDa cut
3	TIA1	iCLIP	hu	GGG_0	single	NNNN_3:6	TG	LUd15.090831.42KYHAAXX.s_6.fq	90-130 kDa cut
1	TIAL1	iCLIP	hu	CA_0	single	NNN_2:4	CC	LUd3.090715.42L2VAAXX.s_7.fq	60-90 kDa cut
1	TIAL1	iCLIP	hu	AG_0	single	NNN_2:4	CC	LUd3.090715.42L2VAAXX.s_7.fq	90-130 kDa cut
1	TIAL1	iCLIP	hu	AC_0	single	NNN_2:4	AT	LUd3.090715.42L2VAAXX.s_7.fq	90-130 kDa cut
2	TIAL1	iCLIP	hu	GA_0	single	NNN_2:4	GA	LUe4.090715.42L7HAAXX.s_2.fq	60-90 kDa cut
2	TIAL1	iCLIP	hu	TG_0	single	NNN_2:4	TG	LUd4.090715.42L7HAAXX.s_1.fq	60-90 kDa cut
2	TIAL1	iCLIP	hu	AC_0	single	NNN_2:4	GA	LUd4.090715.42L7HAAXX.s_1.fq	90-130 kDa cut
2	TIAL1	iCLIP	hu	GT_0	single	NNN_2:4	TG	LUd4.090715.42L7HAAXX.s_1.fq	90-130 kDa cut
3	TIAL1	iCLIP	hu	GCC_0	single	NNNN_3:6	GA	LUd15.090831.42KYHAAXX.s_6.fq	60-90 kDa cut
3	TIAL1	iCLIP	hu	GAA_0	single	NNNN_3:6	GA	LUd15.090831.42KYHAAXX.s_6.fq	90-130 kDa cut
	TIA1	iCLAP	hu	AG_0	single	NNN_2:4	AT	LUd2.090715.42L2VAAXX.s_6.fq	TIA1-C2
	TIA1	iCLAP	hu	AC_0	single	NNN_2:4	CC	LUd2.090715.42L2VAAXX.s_6.fq	TIA1-C2
	TIA1	iCLAP	hu	GTT_0	single	NNNN_3:6	TG	LUd19.090929.42RHUAAXX.s_1.fq	TIA1-C2
	TIA1	iCLAP	hu	GCC_0	single	NNNN_3:6	GA	LUd19.090929.42RHUAAXX.s_1.fq	TIA1-N2
	TIAL1	iCLAP	hu	ATC_0	single	NNNN_3:6	CC	LUd19.090929.42RHUAAXX.s_1.fq	TIAL1-C2
	TIAL1	iCLAP	hu	ACT_0	single	NNNN_3:6	AT	LUd19.090929.42RHUAAXX.s_1.fq	TIAL1-N2
	pG Bead	iCLIP	hu	CA_0	single	NNN_2:4	TG	LUe4.090715.42L7HAAXX.s_2.fq	
	pG Bead	iCLIP	hu	GA_0	single	NNN_2:4	AT	LUmb3.090715.42L2VAAXX.s_5.fq	
