# Supplementary material for: iCLIP Predicts the Dual Splicing Effects of TIA-RNA Interactions
Source: PLoS Biol. 2010 Oct 26;8(10):e1000530. doi: 10.1371/journal.pbio.1000530 (PMC2964331; doi:10.1371/journal.pbio.1000530)
Supplement: Table S2 — Mapping information for iCLIP and iCLAP data. (0.03 MB PDF) [file pbio.1000530.s011.pdf]

Table S2. Mapping Information for iCLIP and iCLAP data.

| | iCLIP | | | | | | iCLAP | | | |
|---|---|---|---|---|---|---|---|---|---|---|
| | TIA1 | | | TIAL1 | | | TIA1 | TIAL1 | Control | |
| | Replicate 1 | Replicate 2 | Replicate 3 | Replicate 1 | Replicate 2 | Replicate 3 | | | Replicate 1 | Replicate 2 |
| Sequence reads matching barcode | 1806192 | 1983312 | 2223993 | 4613203 | 4277738 | 3513826 | 2015615 | 1497290 | 26327 | 477853 |
| Reads mapping to the genome | 1416999 | 1397167 | 1469242 | 3501422 | 3198249 | 2560482 | 791433 | 394465 | 14252 | 85264 |
| Reads after random barcode evaluation | 229928 | 271083 | 368771 | 1255331 | 693096 | 1018374 | 14546 | 2528 | 1074 | 7798 |
| Crosslinking nucleotides | 223785 | 263926 | 351833 | 1207770 | 665624 | 985399 | 14460 | 2507 | 1059 | 7144 |
| Total | | 803354 | | | 2650190 | | 14460 | 2507 | 8203 | |
