# Supplementary material for: iCLIP Predicts the Dual Splicing Effects of TIA-RNA Interactions
Source: PLoS Biol. 2010 Oct 26;8(10):e1000530. doi: 10.1371/journal.pbio.1000530 (PMC2964331; doi:10.1371/journal.pbio.1000530)
Supplement: Table S3 — Enrichment of pentamers surrounding TIA1 and TIAL1 iCLIP and iCLAP crosslink sites. (0.02 MB PDF) [file pbio.1000530.s012.pdf]

Table S3. Enrichment of pentamers surrounding TIA1 and TIAL1 iCLIP and iCLAP crosslink sites.

| kmer | z-score | | | | | | | | | | enrichment | |
|---|---|---|---|---|---|---|---|---|---|---|---|---|
| | TIAL1 | | | | | TIA1 | | | | | TIAL1 | TIA1 |
| | all | 1 | 2 | 3 | iCLAP | all | 1 | 2 | 3 | iCLAP | all | all |
| ATTTT | 714 | 442 | 368 | 344 | 13.5 | 461 | 206 | 255 | 278 | 52.2 | 2.41 | 2.80 |
| TTTTA | 690 | 496 | 407 | 409 | 19.4 | 483 | 291 | 262 | 345 | 53.3 | 2.68 | 3.08 |
| TTTTT | 662 | 558 | 402 | 300 | 12.4 | 378 | 234 | 261 | 225 | 42.0 | 3.21 | 3.37 |
| TTTTC | 628 | 467 | 331 | 294 | 14.0 | 454 | 217 | 276 | 272 | 47.7 | 2.55 | 2.98 |
| TTTAT | 616 | 393 | 302 | 327 | 16.9 | 418 | 229 | 244 | 301 | 59.1 | 2.62 | 3.14 |
| CTTTT | 582 | 410 | 311 | 291 | 16.5 | 403 | 200 | 221 | 236 | 37.7 | 2.40 | 2.79 |
| TTATT | 524 | 347 | 270 | 298 | 16.8 | 350 | 195 | 179 | 267 | 48.9 | 2.51 | 2.96 |
| TATTT | 508 | 358 | 294 | 279 | 14.2 | 395 | 184 | 212 | 276 | 56.5 | 2.36 | 2.76 |
| TTTTG | 435 | 295 | 242 | 191 | 7.3 | 254 | 132 | 177 | 137 | 19.2 | 2.07 | 2.17 |
| TTCTT | 430 | 249 | 228 | 205 | 12.7 | 297 | 171 | 190 | 191 | 35.7 | 2.15 | 2.55 |
| TTTCT | 429 | 309 | 234 | 221 | 15.0 | 361 | 181 | 207 | 185 | 41.4 | 2.18 | 2.57 |
| GTTTT | 421 | 318 | 232 | 196 | 11.1 | 304 | 143 | 163 | 173 | 27.6 | 2.16 | 2.42 |
| TTTAA | 410 | 286 | 252 | 244 | 13.0 | 275 | 136 | 153 | 213 | 32.5 | 2.09 | 2.32 |
| TCTTT | 396 | 312 | 209 | 208 | 11.7 | 293 | 149 | 185 | 181 | 36.7 | 2.17 | 2.56 |
| TGTTT | 382 | 237 | 194 | 170 | 10.0 | 206 | 116 | 143 | 159 | 26.0 | 1.93 | 2.10 |
| TTTAC | 366 | 206 | 156 | 217 | 9.6 | 249 | 123 | 137 | 168 | 30.7 | 2.35 | 2.77 |
| TTTGT | 309 | 212 | 165 | 142 | 8.9 | 193 | 85 | 111 | 108 | 20.2 | 1.84 | 1.92 |
| TTTCA | 307 | 167 | 144 | 181 | 7.4 | 206 | 100 | 107 | 146 | 27.1 | 1.86 | 2.15 |
| ATTTA | 298 | 186 | 120 | 185 | 11.5 | 235 | 99 | 101 | 146 | 31.3 | 1.87 | 2.13 |
| TTGTT | 291 | 190 | 145 | 110 | 10.4 | 165 | 88 | 112 | 104 | 24.7 | 1.86 | 1.98 |
| TTATA | 268 | 179 | 117 | 181 | 11.0 | 193 | 90 | 84 | 170 | 28.1 | 1.92 | 2.17 |
| TTTCC | 263 | 165 | 144 | 127 | 7.9 | 160 | 90 | 102 | 90 | 20.8 | 1.85 | 2.04 |
| CATTT | 254 | 158 | 154 | 146 | 8.0 | 220 | 97 | 118 | 127 | 21.8 | 1.79 | 2.07 |
| AATTT | 248 | 190 | 137 | 149 | 9.1 | 156 | 88 | 99 | 144 | 25.4 | 1.71 | 1.95 |
| TACTT | 242 | 131 | 103 | 128 | 9.9 | 154 | 78 | 92 | 103 | 23.7 | 1.81 | 2.09 |
| ACTTT | 241 | 184 | 133 | 156 | 9.1 | 189 | 92 | 105 | 129 | 23.8 | 1.81 | 2.10 |
| TTCAT | 240 | 130 | 110 | 152 | 6.5 | 201 | 82 | 101 | 122 | 26.6 | 1.83 | 2.13 |
| TTACT | 235 | 145 | 117 | 147 | 8.7 | 170 | 95 | 104 | 115 | 27.2 | 1.98 | 2.30 |
| CTTTA | 233 | 154 | 113 | 152 | 6.0 | 159 | 81 | 82 | 118 | 26.5 | 1.85 | 2.12 |
| TATTA | 228 | 133 | 101 | 124 | 12.8 | 143 | 84 | 61 | 116 | 23.5 | 1.79 | 1.98 |
| ATATT | 223 | 122 | 100 | 147 | 8.8 | 137 | 67 | 87 | 106 | 24.7 | 1.66 | 1.86 |
| CCTTT | 221 | 176 | 127 | 121 | 5.7 | 157 | 94 | 106 | 87 | 19.0 | 1.79 | 2.01 |
| ATTTC | 221 | 118 | 97 | 128 | 8.2 | 151 | 77 | 94 | 109 | 21.7 | 1.72 | 2.02 |
| TTAAT | 218 | 138 | 130 | 131 | 8.8 | 166 | 80 | 94 | 124 | 25.5 | 1.74 | 1.96 |
| TTAAA | 216 | 113 | 90 | 113 | 6.9 | 113 | 64 | 63 | 90 | 17.5 | 1.53 | 1.60 |
| ATTAT | 210 | 132 | 106 | 119 | 12.1 | 158 | 73 | 58 | 109 | 20.2 | 1.73 | 1.92 |
| TCATT | 199 | 117 | 112 | 133 | 4.6 | 155 | 77 | 82 | 109 | 25.2 | 1.72 | 1.97 |
| TTATC | 197 | 110 | 86 | 135 | 4.9 | 180 | 78 | 71 | 95 | 25.5 | 1.94 | 2.24 |
| TTATG | 193 | 117 | 77 | 109 | 5.9 | 122 | 62 | 61 | 86 | 17.7 | 1.72 | 1.91 |
| CTTAT | 183 | 102 | 75 | 113 | 6.4 | 133 | 64 | 72 | 97 | 21.0 | 1.80 | 2.09 |
| CTTTC | 181 | 122 | 86 | 109 | 7.1 | 135 | 82 | 79 | 64 | 18.3 | 1.69 | 1.91 |
| TTCTA | 179 | 112 | 101 | 99 | 9.1 | 126 | 66 | 71 | 82 | 21.0 | 1.70 | 1.89 |
| TAATT | 173 | 124 | 94 | 96 | 5.4 | 123 | 63 | 72 | 90 | 21.2 | 1.57 | 1.74 |
| TCTTA | 168 | 107 | 91 | 106 | 5.3 | 126 | 70 | 70 | 86 | 16.7 | 1.69 | 1.92 |
| GCTTT | 167 | 92 | 88 | 80 | 4.3 | 104 | 60 | 64 | 65 | 9.9 | 1.54 | 1.70 |
| GATTT | 166 | 101 | 105 | 91 | 5.7 | 130 | 63 | 76 | 75 | 14.0 | 1.60 | 1.81 |
| GTTTA | 165 | 111 | 70 | 93 | 6.3 | 123 | 53 | 54 | 79 | 12.5 | 1.64 | 1.81 |
| TTTGA | 165 | 94 | 93 | 69 | 3.4 | 93 | 49 | 61 | 56 | 5.5 | 1.48 | 1.51 |
| TTCCT | 164 | 125 | 83 | 83 | 5.4 | 131 | 63 | 76 | 69 | 18.4 | 1.57 | 1.74 |
| TATTC | 162 | 94 | 79 | 95 | 5.4 | 115 | 65 | 61 | 72 | 21.4 | 1.67 | 1.87 |
