# Supplementary material for: iCLIP Predicts the Dual Splicing Effects of TIA-RNA Interactions
Source: PLoS Biol. 2010 Oct 26;8(10):e1000530. doi: 10.1371/journal.pbio.1000530 (PMC2964331; doi:10.1371/journal.pbio.1000530)
Supplement: Table S4 — RT-PCR primers for validation of splicing event predicted by the microarray. (0.08 MB PDF) [file pbio.1000530.s013.pdf]

Table S4. RT-PCR primers for validation of splicing event predicted by the microarray.

Gene Symbol	Gene Description	Exon	Included Region	Excluded Region	Strand	dI (%) Array	dI (%) PCR	Forward Primer	Reverse Primer	Product Size
**Cassette Exons**										
ACACA	Acetyl-CoA carboxylase 1	E38	chr17:32641494-32641517	chr17:32638827-32652758	-	28.80	59.03	ACAACACCTGTGTGGTGGAA	TGTCCAACAGTACATCGCTGA	153(in)/129(ex)
ANLN	Actin-binding protein anillin	E13	chr7:36423214-36423324	chr7:36422018-36425378	+	43.05	32.51	GAAAAGGTGACCGAAAACCA	CTCCTCTTGGCTCTTCTCCA	291(in)/180(ex)
ASPH	Aspartyl/asparaginyl beta-hydroxylase	E17	chr8:62721863-62721991	chr8:62719745-62726163	-	20.53	12.58	AAGAGGCTGAGCCACACACT	CTTCCACGTGGTAACTATGCTC	282(in)/153(ex)
C5orf44	UPF0533 protein C5orf44	E9	chr5:64987219-64987236	chr5:64984128-64989951	+	52.78	56.43	TCAAACCATTGGATGTGAAAA	TGGCTCCAGTGAAACCTTCT	140(in)/122(ex)
CAST	Calpastatin	E3	chr5:96024485-96024626	chr5:96023625-96036999	+	-31.20	-5.24	CCTCCCCGCCACTCTC; GCCTCCAACGCAACACC	GTTTGGAAGGCGATTCACTG	138(in)/169(ex)
FGF5	Fibroblast growth factor 5 Precursor	E3	chr4:81415087-81415190	chr4:81407357-81426503	+	53.94	13.77	TCTACTGCAGAGTGGGCATC	CTTGAAAACGCTCCCTGAAC	231(in)/127(ex)
FOXP2	Forkhead box protein P2	E2	chr7:113590235-113590379	chr7:113513902-113715310	+	22.10	23.61	TGCCACCTCCCGGGACGC	TGTTTCTTCCAGATCTTCCACA	276(in)/137(ex)
FYN	Proto-oncogene tyrosine-protein kinase Fyn	E16	chr6:112127412-112127566	chr6:112124353-112128000	-	-16.81	-26.56	GGCCCAGTTTGAAACACTTC	CACCACTGCATAGAGCTGGA	337(in)/346(ex)
HNRNPC	Heterogeneous nuclear ribonucleoproteins C1/C2	E4	chr14:20801310-20801335	chr14:20772228-20807297	-	26.73	51.03	GCAGCAGTCGGCTTCTCTAC	CCCAATGAATACACGGGAGT	200(in)/174(ex)
hsa-mir-16-1	hsa-mir-16-1	E7	chr13:49521256-49521326	chr13:49517369-49521721	-	39.73	3.34806167	TTGAAAGGTGTACTGCAAGGAA	TGCCAACTTAACAGGGCAAT; AAAACTTTTTCCATGAGGAAGG	168(in)/218(ex)/91(ex)
LIMCH1	LIM and calponin homology domains-containing protein 1	E21	chr4:41335706-41335741	chr4:41316214-41341274	+	-13.5	-3.25078	GAGGAATACCGCAAGAGCTG	CTTGCCATTTGTCGTCCTCT	223(in)/187(ex)
LRRFIP2	Leucine-rich repeat flightless-interacting protein 2	E24	chr3:37107963-37108033	chr3:37100302-37111287	-	-13.31	-22.17	CCTCAGCAACAACCCCTCTA	TTCTGCCATCTGTTCCTCCT	311(in)/239(ex)
KYNU	Kynureninase	E16	chr2:143462884-143462943	chr2:143460060-143503666	+	-27.8	no change	TCATGAAAAGCATGCCCATA	TGGTTCTCGGCTACATACTGG; AGACCCCAGGGATTAACTGC	154(in)/110(ex)
LSM14B	LSM14 protein homolog B	E6	chr20:60136036-60136152	chr20:60134890-60138236	+	-16.17	12.7334783	ACGTGCCTTACAGCCCTTT	AGCATTCAGGTTGTCAGCAG; GGCTGTGGAGATGGAGAAGA	182(in)/163(ex)
MAPK14	Mitogen-activated protein kinase 14	E10	chr6:36175943-36176022	chr6:36171821-36178326	+	-23.13	-11.27	TTGGTCAGTGGGATGCATAA	TGGGGTTCCAACGAGTCTTA; GGCTTGGCATCCTGTTAATG	140(in)/109(ex)
MAST2	Microtubule-associated serine/threonine-protein kinase 2	E11	chr1:46239218-46239238	chr1:46236058-46241064	+	-24.70	-42.30	CCCGGAATTTCTCTCCAAAT	CCTGAAGAGGGCAAAGAGG	121(in)/100(ex)

MCM8	DNA replication licensing factor MCM8	E14	chr20:5901223-5901372	chr20:5900684-5901702	+	-13.44	-17.30	TTTGCCCTGTCATTTTTGGT; CTCCACCTCCCAAGTTCAAA	CTAGGCCTGGATCTCCAACA	211(in)/147(ex)
MED31	Mediator of RNA polymerase II transcription subunit 31	E3	chr17:6494026-6494122	chr17:6488703-6494400	-	35.95	34.15	TCGGTTTCAGTTGGAGTTGG	GCTCCTTTCGGAAGTGTTCA	215(in)/118(ex)
MLL5	Histone-lysine N-methyltransferase MLL5	E3	chr7:104465809-104465882	chr7:104442218-104468522	+	21.68	6.55	GAGGTGCGGAGAGACTCCTT	GTTCGCCTCTGGTAAATTCG	256(in)/182(ex)
MRPS18C	28S ribosomal protein S18c, mitochondrial Precursor	E5	chr4:84598523-84598606	chr4:84597135-84599917	+	51.95	43.96	TTCACAACAGGTATCCAGCAA	TGCCTTCCATAAATGCATCC	165(in)/81(ex)
NF1	Neurofibromin	E31	chr17:26604082-26604144	chr17:26600263-26609488	+	23.48	21.22	CGAAGTGTGTGCCACTGTTT	ACTTCAAGCCCCTTTCGATT	238(in)/175(ex)
OCRL	Inositol polyphosphate 5-phosphatase OCRL-1	E21	chrX:128546003-128546025	chrX:128538211-128548660	+	-24.93	-24.48	TGTGCCGTATGAAAAGACCA	GGAACCTGAAGGGGTCTCTC	145(in)/121(ex)
OGT	UDP-N-acetylglucosamine--peptide N-acetylglucosaminyltransferase 110 kDa subunit	E9	chrX:70691765-70691960	chrX:70691167-70692529	+	37.07	21.07	CCCTTGACCCAAACTTTCTG	GGACACAGACGGAGAGCTGTA	316(in)/120(ex)
PLD1	Phospholipase D1	E18	chr3:172887169-172887282	chr3:172878178-172887855	-	-38.62	-22.21	ATCAGCAGCATTGACAGCAC	GTCCTTTCCATGCCAGAATC	216(in)/106(ex)
PON2	Serum paraoxonase/arylesterase 2	E11	chr7:94874235-94874316	chr7:94873495-94877149	-	32.12	22.89	TGATTCAGCAAATGGGATCA	GCTTCTGGCCATTAGGATGA	212(in)/130(ex)
PRPF40A	Pre-mRNA-processing factor 40 homolog A	E10	chr2:153243816-153243869	chr2:153242235-153244112	-	43.15	56.16	AATGCCCCTGGAAGGAATAC	TTGTGGTGCACTCTTCTTGC	210(in)/156(ex)
SEMA3B	Semaphorin-3B Precursor	E12	chr3:50286194-50286263	chr3:50286081-50286798	+	-33.70	-8.01	ACACCCACTTCGATCAGCTC	TTGTACATGAGGGGGTGGTT	325(in)/113(ex)
SS18	SSXT protein	E16	chr18:21869793-21869885	chr18:21869089-21872517	-	-28.12	-17.31	GTGGACAAGGTCCTCCAGAA	GGGTGGATATCCCTGTTGTG	209(in)/116(ex)
SSFA2	Sperm-specific antigen 2	E18	chr2:182493569-182493622	chr2:182492418-182494920	+	44.08	32.70	TCCGAATGGAACTTCAGGAC	CTGCTCCTGCATCAGTTCAG	203(in)/137(ex)
UBAP2	Ubiquitin-associated protein 2	E12	chr9:33963181-33963235	chr9:33953789-33976758	-	31.97	32.00	TGCAATCAAGTGGACAAACC	TTTTGTTTGACAGATCCTGAGC	259(in)/100(ex)

Alternative 5' splice site

ADAR	Double-stranded RNA-specific adenosine deaminase	E13	chr1:152829284-152829362	chr1:152829028-152829362	-	30.51	28.13	GTCTTGATTGGGGAGAACGA	GGGCTGGAAGCTGTTAGTCA	213(in)/135(ex)
CCNT2	Cyclin-T2	E9	chr2:135420186-135420505	chr2:135420186-135421776	+	-15.48	-15.53	GGATTTGGCACAGACATCCT; CAGAGGGGTTAAATGCACAA	TATTCCCACCAATGCTTTCC	278(in)/168(ex)

CHD9	Chromodomain-helicase-DNA-binding protein 9	E39	chr16:51899318-51899366	chr16:51899318-51900099	+	30.95	39.59	GGTGCCGGTGTTAAAGAAGA	TCGAAGCCGAGTGAGGTACT	204(in)/156(ex)
CLIP4	CAP-Gly domain-containing linker protein 4	E17	chr2:29233717-29233772	chr2:29233717-29236703	+	-31.24	-25.78	CAAAAGCCCTTCCCTTTCAT	AATGGTGCCCAGTCTCTGTC	194(in)/139(ex)
FNTA	Protein farnesyltransferase/geranylgeranyltransferase type-1 subunit alpha	E8	chr8:43051664-43054741	chr8:43051664-43057418	+	-22.65	-10.88	AGCTGCAGTATGTGGACCAA	CCCAGTGCCTGAGATATGGT; CCATGCACTTTCATTATGTGG	198(in)/176(ex)
MARCH	E3 ubiquitin-protein ligase MARCH7	E9	chr2:160324092-160324115	chr2:160324092-160327637	+	-11.99	-12.88	TTGGAGCTTAACCTGGAGGA	CGCACAAGTGCAATAACACC	144(in)/121(ex)
MYNN	Myoneurin	E4	chr3:170974913-170975043	chr3:170974913-170979250	+	23.07	26.06	TGATTGGGGAATTCCAGTTT	TGACCACCTCTTCCACTTTG	241(in)/110(ex)
**Alternative 3' splice site**										
C3orf23	Uncharacterized protein C3orf23	E8	chr3:44383953-44384028	chr3:44378015-44384028	+	-15.91	-31.18	AGACCAGAGTTCCTCCGATG	TCATCAGGGTCCTTGAATCC	258(in)/182(ex)
CLINT1	Clathrin interactor 1	E12	chr5:157149100-157149153	chr5:157149100-157151289	-	-17.96	-26.72	CCTGCTGCCTCAAATTCTTC	TAGAGGGCAAGGTTTTGCTG	238(in)/184(ex)
PDSS1	Decaprenyl-diphosphate synthase subunit 1	E7	chr10:27049071-27049153	chr10:27038704-27049153	+	-15.83	-19.59	TTGATGGGAAAGGGAAAGC	TTCGAGAACTtgCATCGTCA	266(in)183(ex)
TBCE	Tubulin-specific chaperone E	E8	chr1:233649356-233649411	chr1:233644557-233649411	+	-14.16	no change	CCCGACAGGAGGATCCTTTA	TGCTTCAGCAACTCCTCCTT	321(in)/265(ex)
**Predicted alternative 5' splice site**										
C9orf97	Uncharacterized protein C9orf97	E4	chr9:99429410-99429501	chr9:99428101-99429501	-	-2.85	-21.99	GACCAAGGAGATGACCTGGA	TTTGACAAAAAGGGCAAAGG	257(in)/165(ex)
PCMT1	Protein-L-isoaspartate	E12	chr6:150165199-150165245	chr6:150165199-150173445	+	-32.05	-38.06	GCTACAAGATGGCAGCATCA	CAAGCTGTGATGGTGTTGGT	234(in)/187(ex)
PRPF38B	Pre-mRNA-splicing factor 38B	E4	chr1:109039916-109039925	chr1:109039916-109040188	+	2.09	4.60	ACGTTGAACCATGGGAGAAA	CCCATCACTTGCTTTCGAGT	167(in)/157(ex)
UPF1	Regulator of nonsense transcripts 1	E8	chr19:18824881-18824913	chr19:18824881-18825061	+	4.21	3.34	CCTGGGCCTTAACAAGAAGA	TGTACCGCAGGCATATCTCA	132(in)/98(ex)
WHSC1L1	Histone-lysine N-methyltransferase NSD3	E32	chr8:38258143-38258175	chr8:38256357-38258175	-	-23.66	-26.88	GAATGCAGATTGCGAATCAA	AGTCCCACTCGAACATCTCC	188(in)/155(ex)
