# Supplementary material for: iCLIP Predicts the Dual Splicing Effects of TIA-RNA Interactions
Source: PLoS Biol. 2010 Oct 26;8(10):e1000530. doi: 10.1371/journal.pbio.1000530 (PMC2964331; doi:10.1371/journal.pbio.1000530)
Supplement: Table S5 — qPCR primers for validation of intron retention event. (0.06 MB PDF) [file pbio.1000530.s014.pdf]

Table S5. qPCR primers for validation of intron retention event.

Gene Symbol	Gene Description	Exon	Included Region	Excluded Region	Strand	Array Predicton	qPCR fold change (Control as 0)	Forward Primer	Reverse Primer	Forward Primer (within exon)	Reverse Primer (within exon)
ARL16	ADP-ribosylation factor-like protein 16	E4	chr17:77260562-77260971	chr17:77260562-77260971	-	dI negative	2.9655	GATGGGAAAGGCGACCTG	CCAAGCGCAGAACTGACC	GATGGGAAAGGCGACCTG	TCTTTCTCTGTGCCACGATG
CDC7	Cell division cycle 7-related protein kinase	E16	chr1:91751453-91752093	chr1:91751453-91752093	+	dI negative	1.7174	TCAGCAGTCCACCACAAAAG	CCCTCTGTTGGTTGAAGGAG	GCTCAGCAGGAAAGGTGTTC	AGCTTTTGTGGTGGACTGCT
DNAJC4	DnaJ homolog subfamily C member 4	E6	chr11:63756013-63756478	chr11:63756013-63756478	+	dI positive	-1.1321	TGCCAGCACTGAGGAAGTTA	CTGGCTCAAAGTTCCTGCTC	ATGAACTGTTGGGGGTGCAT	TTGGACTTGGAGAAGAAAGCTC
EIF2B1	Translation initiation factor eIF-2B subunit alpha	E8	chr12:122677332-122677540	chr12:122676994-122677544	-	dI negative	1.9655	GGCCAAGAAGCGATTTAGTG	GGGCACATTCTGGTTCTGAT	CCTGAGAGTCCTGGAAGCAG	CCTGACAAATCAGGCTGTGA
ELMOD2	ELMO domain-containing protein 2	E3	chr4:141664885-141666024	chr4:141664885-141666024	+	dI negative	1.2116	CTCCTGCTCCCCCTAGTTCT	GGGGCAAGAGGAAAAGAAAC	GGTGCTTGAAGGGAGTGTTC	AGAACTAGGGGGAGCAGGAG
NCBP1	Nuclear cap-binding protein subunit 1	E29	chr9:99474885-99475615	chr9:99474885-99475615	+	dI positive	-0.5281	AGCCTGCTCAACGTGAAGAT	CCCAGCCCATATGCTATATTCT	TTTCTTCCACCCCTGAGATG	AGCAGGCTGAGGAAGACAAG
NCBP2	Nuclear cap-binding protein subunit 2	E8	chr3:198148917-198150519	chr3:198148917-198150519	-	dI negative	0.7400	TCTTCAGCAAAAGTGGTGACA	CCCTGGTTCAGCAGCTACAT	TCTTCAGCAAAAGTGGTGACA	CACAAAACAGAATCCACATGC
NISCH	Nischarin (Imidazoline receptor 1)	E15	chr3:52489352-52489935	chr3:52489352-52489935	+	dI positive	-1.1463	CAGCCCATCCTCTCTAACCA	TGTGTCTGACCCTGTTGGAA	GTGAAGACTCCCGGCTCTC	GGAGCTGCTGGGTCTGATG
PAPOLA	Poly(A) polymerase alpha	E15	chr14:96070660-96070979	chr14:96070654-96071969	+	dI negative	0.8977	GGCCAAATCCAGTGCTATTG	CCCTGGAGTTTATGCAGATGA	GGCCAAATCCAGTGCTATTG	GGGTCCCATACAGGCAAAT
PKN2	Serine/threonine-protein kinase N2	E13	chr1:89043943-89044162	chr1:89043943-89044162	+	dI negative	0.5749	GTGCCAGTGGTTGATGTACG	AAGCAAGACTCAGTACACCTGCT	CCTCAAGCTCCTGTGCCTAC	GCTGGAGGTGCTAGTTGAGG
PPIA	Peptidyl-prolyl cis-trans isomerase A	E6	chr7:44805605-44805826	chr7:44805605-44805826	+	dI negative	0.9400	AGGGTTCCTGCTTTCACAGA	TGGCTCAATCCCAAATGAAG	GCTCTGAGCACTGGAGAGAAA	CTGTGAAAGCAGGAACCCTTA
PYCR2	Pyrroline-5-carboxylate reductase 2	E5	chr1:224176403-224176527	chr1:224176403-224176527	-	dI positive	-1.3151	CACCATCAGCTCTGTGGAGA	AGGGAGAGAGCCGAATGAG	AGACACATCGTGGTCTCCTG	CTCCACAGAGCTGATGGTGA
RPUSD3	RNA pseudouridylate synthase domain-containing protein 3	E9	chr3:9857490-9858667	chr3:9857490-9858667	-	dI positive	0.5829	CTGCCAGCTGAGAACAACAA	CAGTAGTCCCAAGCTGCACA	ACATGTACTCTGCCCGTGTG	CTGTCTTTGGGGCTTGTTGT
SFRS12	Splicing factor, arginine/serine-rich 12	E14	chr5:65495507-65496192	chr5:65495507-65496192	+	dI positive	-0.6952	GAAGCGAGTACGAGAAGCTCA	TGAAGTGACTGAACAAGATGATTTC	CAGGCTGCAGCTAAGGAGTT	TGAGCTTCTCGTACTCGCTTC
SFRS18	Splicing factor, arginine/serine-rich 18	E26	chr6:99954925-99955141	chr6:99954909-99955141	-	dI positive	-0.3265	GGTCTGGTAAGAAGGCAAGC	GAGAGAGAGAAAGGACACTTTCAA	CGTGGAGAAATCTCAAAGGTC	AGACTTGTGTTTGCGGCTTG
SFRS18	Splicing factor, arginine/serine-rich 18	E3	chr6:99970345-99970946	chr6:99970345-99970946	-	dI positive	-2.6816	AATCTTGCTGCTATGCTTCG	CCAAGAAGACACACCAACTGC	AATCTTGCTGCTATGCTTCG	CTCAGAGGTTCACCTTCTGTTT
SRRM1	Serine/arginine repetitive matrix protein 1	E5	chr1:24845868-24846157	chr1:24845868-24846157	+	dI positive	-0.5954	TCCTTGGGTTTGAAGATGATG	TTCAGAAACAAGAGCCATTTG	TCCTTGGGTTTGAAGATGATG	TTCACTTCCAGCTGGTTGAAT
ZDHHC13	Probable palmitoyltransferase ZDHHC13	E17	chr11:19142487-19143175	chr11:19142487-19143175	+	dI negative	1.2580	ATTTGTGCCAATGCCTCTGT	TGCATACATGGCAGTGGAGT	CTGCCATGTATGCAACTGCT	GTCCAGTCCACAGGCAGTGT
