# Supplementary material for: iCLIP Predicts the Dual Splicing Effects of TIA-RNA Interactions
Source: PLoS Biol. 2010 Oct 26;8(10):e1000530. doi: 10.1371/journal.pbio.1000530 (PMC2964331; doi:10.1371/journal.pbio.1000530)
Supplement: Text S1 — Supplementary methods. A detailed explanation of the iCLIP, iCLAP, z score analysis, identification of significant iCLIP crosslink sites, and RT-PCR analysis is provided. (0.04 MB DOC) [file pbio.1000530.s015.doc]

**iCLIP**

HeLa cells were UV crosslinked before lysing in lysis buffer (50mM Tris-HCL pH7.4; 100mM NaCl; 1% NP-40; 0.1% SDS; 0.5% sodium deoxycholate and proteinase inhibitor (Calbiochem)) and sonicated. The lysates were split and treated with Turbo DNAse I (Ambion) and High (1:50 dilution) or Low (1:3000 dilution) of RNAse I (Ambion). Protein G Dynabeads (Invitrogen) conjugated to goat-anti TIA1 antibody (Santa Cruz, C-20) or goat-anti TIAL1 antibody (Santa Cruz, C-18) was used for immunoprecipitation. After immunoprecipitation, Dynabeads were washed with high-salt buffer (50mM Tris-HCl pH7.4; 1M NaCl; 1mM EDTA; 1% NP-40; 0.1% SDS; 0.5% sodium deoxycholate) and PNK buffer (20mM Tris-HCl pH7.4; 10mM MgCl_2_; 0.2% Tween-20). All the High and 1/5 of Low samples were labelled with ^32^P-γ-ATP at 37°C for 5 min with PNK (NEB). The rest of the Low samples were first dephosphorylated with Shrimp Alkaline Phosphotase (Promega) at 37°C for 10 min, then ligated to 3’ adaptor using T4 RNA ligase (NEB) and 25% PEG-400 at 16°C overnight (L3 adaptor: 5’-phosphate-Barcode-AGAUCGGAAGAGCGGTTCAG-3’-Puromycin). For barcode on both L3 and L5 adaptor, see Supplementary Table 1. After ligation, all samples were eluted with 1xNuPAGE Sample Buffer (Invitrogen). The eluate were loaded onto 4-12% NuPAGE SDS gels (Invitrogen) and transferred to nitrocellulose membranes.

The radiogram were visualised and the protein-RNA complexes corresponding to the RNA size 50-200 nt were cut out from the membrane, and digested with proteinase K (NEB) at 55°C for 30 min in 200μl PK buffer (100mM Tris-HCl pH7.4, 50mM NaCl, 10mM EDTA) followed by adding 140μl PK/7M urea buffer (100mM Tris-HCl pH7.4, 50mM NaCl, 10mM EDTA, 7M urea) at 55°C for 30 min. The RNA was then extracted by phenol/chloroform purification, and precipitated before being reverse transcribed using superscript III (Invitrogen). The RT primer had regions complimentary to the 3’ adaptor together with the 5’ adaptor for Solexa sequencing separated by BamHI digestion site (5’-phosphate-NNN-Barcode-AGATCGGAAGAGCGTCGTGgatcCTGAACCGC-3’). The resulting cDNA was then gel purified using 6% TBU gels (Invitrogen). Sizes corresponding to 50-100 nt and 100-200 nt cDNA was cut out from the gels, extracted by incubating at 37°C for 2 hours in TE buffer (50mM Tris-HCl pH7.4; 1mM EDTA) and precipitated. The cDNA was then self-ligated using CircLigaseII (Epicentre Biotechnologies, CL9025K) at 60°C for 1 hour. A primer that is complimentary to the BamHI sites was then annealed to the circular cDNA (5’-GTTCAGGATCCACGACGCTCTTCAAAA-3’) and the cDNA was re-linearised by digesting with BamHI. The resulting cDNA had 3’ adaptor and 5’ adaptor at the either site, respectively. The products were then amplified by PCR using primers compatible with Solexa sequencing (5’-CAAGCAGAAGACGGCATACGAGATCGGTCTCGGCATTCCTGCTGAACCGCTCTTCCGATCT-3’; 5’-AATGATACGGCGACCACCGAGATCTACACTCTTTCCCTACACGACGCTCTTCCGATCT-3’, oligonucleotide sequences © 2006 and 2008 Illumina, Inc. All rights reserved). They were then visualised on 6% TBU gels before sequenced with 54 cycles on Illumina GA2 single-end sequencing.

**iCLAP**

Human TIA1b and TIAL1b isoforms were PCR amplified and cloned into a pcDNA3 vector containing an expression cassette with N-terminal or C-terminal Strep and His tags. Plasmids were transfected using polyfect (Qiagen) according to manufacturers’ instructions. The cells were crosslinked 2 days after transfection. The cells were lysed in lysis buffer (50mM Tris-HCl pH7.4; 100mM NaCl; 0.1% NP-40) and sonicated. After DNase and RNase I digestion, M280 beads (Invitrogen) were used to first purifiy the protein-RNA complex via Strep tags. The beads were washed with high-salt buffer (50mM Tris-HCl pH7.4; 1M NaCl; 0.1% NP-40) and PNK buffer. The protein-RNA complex bound to beads was then subjected to ^32^P-γ-ATP labelling or 3’ adaptor ligation as described above. The samples were eluted from the bead by 100μl elution buffer (50mM Tris-HCl pH7.4; 100mM NaCl; 8M urea, 0.1% SDS) at 37°C for 5 min. The eluate were diluted up to 1 ml with lysis buffer, and further purified by cobalt bead (Thermo Scientific) via His tag. The protein-RNA complex was eluted and the remaining procedure was the same as for iCLIP.

**Pentamer z-score analysis**

(i) iCLIP reads were associated with expressed genomic regions as defined by ENSEMBL (version Hg18/NCBI36). Each coding or non-coding gene was defined as its own region (in case of overlapping genes, the shorter gene always had the priority). Introns, 5’ UTR, ORF and 3’ UTR were considered as separate regions. (ii) iCLIP reads antisense to the transcriptional direction of the associated gene and reads that mapped to non-annotated genomic regions were removed before proceeding to further analysis.  (iii) The control files were generated 100 times with randomised iCLIP positions. iv) Both in iCLIP and control files, the positions were extended by 10 nt in both directions, such that 21 nt long sequences were used for analysis. v) The occurrence (pentamer frequency) was calculated for each pentamer in each file. vi) The z-score was calculated for each pentamer as:

(occurrence in iCLIP sequences – average occurrence in control sequences) / standard deviation of occurrence in control sequences

**Nucleotide representation of the RNA motifs**

To calculate base frequencies of iCLIP sequence reads, we extracted 21 nt of genomic sequence surrounding each significant crosslink site (FDR<0.05). Graphic representation of nucleotide composition at -10 to +10 positions relative to the crosslink site (position 0) was generated using Weblogo 3 (http://weblogo.berkeley.edu).

**Identification of significant iCLIP crosslink sites**

This followed the same statistical approach as the analysis of CLIP sequence clusters [42] with a few modifications. (i) iCLIP reads were associated with expressed genomic regions as defined by ENSEMBL hg18 release of human genome. Both coding and non-coding genes were included (in case of overlapping genes, the shorter gene always has the priority). Introns, 5’ UTR, ORF and 3’ UTR were considered as separate regions. (ii) iCLIP reads antisense to the transcriptional direction of the associated gene, and reads that mapped to non-annotated genomic regions, were removed before proceeding to further analysis.  (iii) Control file with random placement of iCLIP reads on corresponding genes was generated 100 times. Each 5’UTR, 3’ UTR, and each intron is its own region; all remaining parts of the gene are its own region (these will be all exononic sequences corresponding to ORF). (iv) To identify significant crosslink positions, cDNA values in iCLIP or randomised positions were summed for positions up to 15 nt apart, and the resulting values were considered the ‘height’ of each crosslink site. (v) For a particular height, h, the associated probability of observing a height of at least h was Ph = Σ ni(i = h:H)/N. (vi) The modified FDR for a peak height was computed as FDR(h) = (muh + sigmah)/Ph, where muh and sigmah is the average and s.d., respectively, of Ph,random across the 100 iterations. (vi) Within each region of a gene (intron, 5’ UTR, 3’UTR, ORF, ncRNA), the smallest height that gave an FDR < 0.05 was defined as the threshold height (h*). Crosslink sites at positions satisfying h > h* were considered significant.

**RT-PCR**

Total RNA was extracted using RNasy Kit (Qiagen) and 200ng of RNA was used for reverse transcription using Superscript II (Invitrogen) according to the manufacturers’ instruction. Real-time PCR was performed using SybrGreen (Applied Biosystems) with 50 nM primer concentration. The data were collected as absolute Ct values and the relative expression levels were calculated. For analysis of intron retention, qPCR was performed using primers listed in Table S5.

For analysis of splicing changes, PCR was performed using Immomix (Bioline) using primers listed in Table S2. The PCR products were visualised using QIAxcel capillary electrophoresis system (Qiagen). The signal peaks were calculated by using the normalised area of the peaks divided by their molecular size. Percentage of exon inclusion was determined as the signal of the inclusion isoform divided by the sum of signals for inclusion and exclusion isoforms.
